# Substituent Effect versus Aromaticity—A Curious Case of Fulvene Derivatives

**DOI:** 10.1021/acs.joc.3c01539

**Published:** 2023-09-29

**Authors:** Pawel A. Wieczorkiewicz, Krzysztof K. Zborowski, Tadeusz M. Krygowski, Halina Szatylowicz

**Affiliations:** † Faculty of Chemistry, Warsaw University of Technology, Noakowskiego 3, Warsaw 00-664, Poland; ‡ Faculty of Chemistry, Jagiellonian University in Kraków, Gronostajowa 2, Kraków 30-387, Poland; § Department of Chemistry, University of Warsaw, Pasteura 1, Warsaw 02-093, Poland

## Abstract

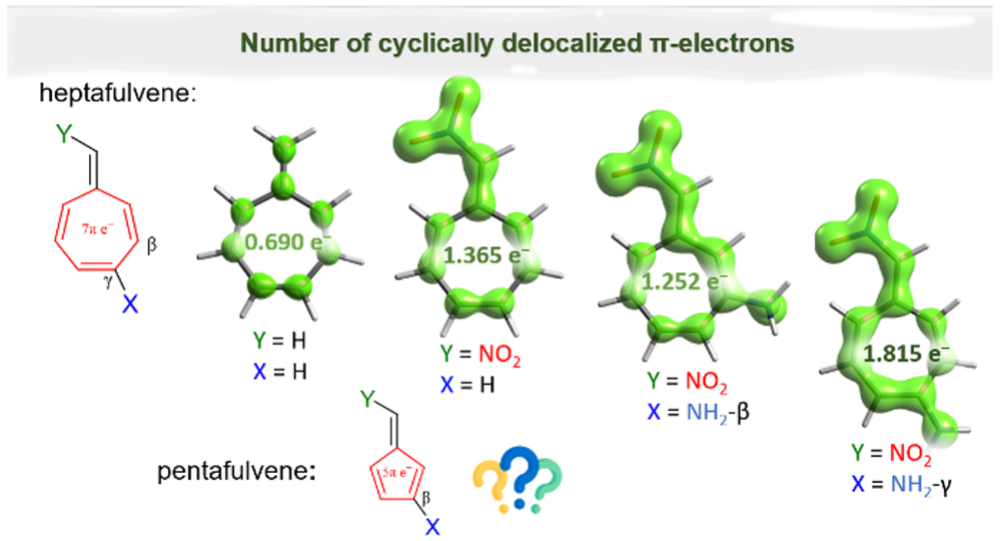

A computational study on amino- and nitro-substituted penta- and heptafulvenes reveals the interplay between the aromaticity and the substituent effect (SE). Ring substitution alone has little influence on the aromaticity, but in combination with an exo substituent of opposite properties, it substantially enhances the cyclic π-electron delocalization. Despite the SE being stronger for β substitution, only γ substitution leads to higher aromaticity. An explanation is provided by the electron density of delocalized bonds (EDDB) method, which proves to be a valuable tool in analyzing both cyclic delocalization and the SE.

Fulvenes form an important class of organic compounds. Their two most common representatives are pentafulvene (5-methylenecyclopenta-1,3-diene) and heptafulvene (7-methylenecyclohepta-1,3,5-triene). Due to their specific reactivity, most importantly in cycloaddition reactions,^1,2^ fulvenes appear in many branches of organic synthesis, often as a precursor of polycyclic compounds.^3^ In coordination chemistry, pentafulvenes offer many sites for binding with metal centers, with η^2^, η^4^, η^6^, and η^5^:η^1^ complexes reported in the literature, or are important reactants in the synthesis of cyclopentadienyl and carbene complexes. Among them are complexes with Ti,^4^ Nb,^5^ Zr,^6^ and Re^7^ metal centers, some with potentially interesting applications. Heptafulvene complexes are less common.^8^ A novel field in fulvene chemistry is the development of photochromic dyes;^9^ some fulvene derivatives show interesting electrochromic behavior as well as polymers functionalized with penta- and heptafulvenyl units.^10^ It is also worth mentioning that heteropentafulvenes with one or more endocyclic C atoms replaced by B, Si, or P have been synthesized and characterized.^11^ For a review summarizing recent developments and applications of pentafulvene chemistry, readers are referred to ref (12).

Moreover, fulvenes are an important object of theoretical research. In general, these compounds are best characterized as olefinic rather than aromatic, albeit it has been shown that under some circumstances the exocyclic π-electron density can be pumped into or out of the penta/heptacycle, which, in turn, switches the character of the molecule between aromatic (according to Hückel’s rule 6 π ring electrons) and antiaromatic (4 or 8 π electrons) states. Depending on factors such as exo substitution or complexation, they can approach some characteristics of aromatic compounds, such as stability, bond equalization, and diatropic ring current.^13^ Therefore, fulvenes are useful model compounds for theoretical studies of aromaticity and substituent effects. For example, exocyclic substitution with an electron-donating group increases the aromaticity of pentafulvene, while an electron-withdrawing group decreases it, as illustrated by the resonance structures for the amino and nitro derivatives in Scheme 1. This phenomenon has been observed both in the crystallographic data^14^ and from DFT calculations.^15^ The latter show good correlations between aromaticity indices and Hammett constants of the exo substituents.^16^ In a similar way, complexation of pentafulvene with an alkali metal atom^17^ increases its aromaticity, while in heptafulvene, the same can be achieved with the halogen atom.^18^ Substituent effects on the aromaticity of fulvenes also influence their excited state properties.^19^ It has been reported that exo- and endocyclic substituents can modify the Hückel (6 π electrons in the lowest singlet state) and Baird (4 π electrons in the lowest triplet state) aromaticity of fulvene. Consequently, this allows one to tune the singlet–triplet energy gap by stabilizing or destabilizing singlet or triplet states.^20^ Such approach can be useful, for example, in designing new chromophores for singlet fission materials.^21^

**Scheme 1:**
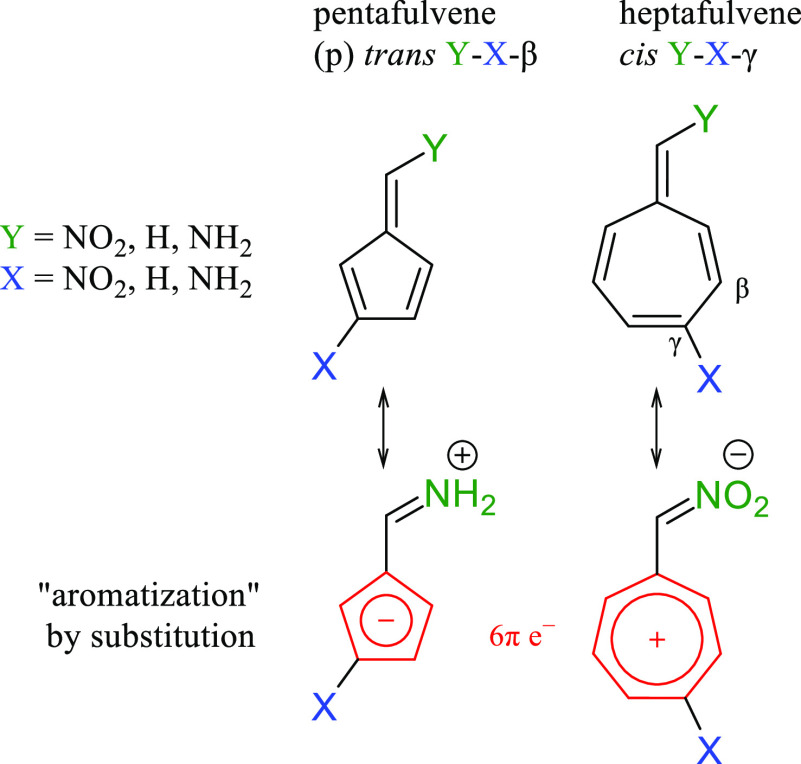
Studied Fulvene Derivatives and Notation Used Throughout This Paper (for details see Figure S1) All combinations of Y and X substituents (X in the β or γ position) and cis/trans isomers were analyzed.

Due to the interesting properties of fulvenes, the interaction between two substituents in the systems shown in Scheme 1 is studied to find out how this interaction influences the cyclic π-electron delocalization using aromaticity indices and EDDB visualization.^22^ As substituents, NH_2_ and NO_2_ groups were selected, representing strongly electron-donating and -withdrawing groups with Hammett constants σp = −0.66 and 0.78, respectively.^23^Figure 1 presents the values of three aromaticity indices calculated for the studied systems: HOMA, FLU, and EDDB_P_(π). Additionally, in Table S2, the aromatic stabilization energies (ASE) and NICS(1)_*zz*_ values are presented; they generally show similar trends to HOMA, FLU, and EDDB_P_(π). In the case of NICS, the values depend on the ring size.^24^ In Figure 1, the systems are grouped into five classes, according to their structural similarity. The most aromatic are hepta Y = NO_2_ and penta Y = NH_2_, X = NO_2_ systems. The second group, with lower HOMA values, consists of two classes of compounds: penta Y = NH_2_, X = H, NH_2_ and hepta Y = H, NH_2_. The third group, with negative values of HOMA, contains only pentafulvenes with Y = H or NO_2_. This sequence is in agreement with the π-electron deficit in pentafulvene and excess in heptafulvene rings and the electronic properties of the Y = NH_2_ and NO_2_ groups. An electron-donating or -withdrawing exocyclic Y group significantly increases the aromaticity of the penta- or heptafulvene ring, respectively. By contrast, ring substitution at the β or γ position (X group) has little effect on the aromaticity. Differences in aromaticity are reflected in the stability of particular derivatives—the most stable isomers of nitroheptafulvene and aminopentafulvene are substituted in the exocyclic position (data on the stabilities of all compounds are presented in Table S1). It should be mentioned that heptafulvenes belonging to the most aromatic Y = NO_2_ class (Figure 1) are all planar, whereas all Y = NH_2_ derivatives are not planar (Table S2), which is associated with their lower aromatic character. A combination of Y and X groups with opposite electronic properties tends to increase aromaticity. It can be noticed in the case of pentafulvene (p) cis/trans NH_2_–NO_2_-β and heptafulvene cis/trans NO_2_–NH_2_-γ derivatives. The aromaticity of their rings is higher in comparison to the monosubstituted (X = H) or disubstituted systems with the X group being the same as Y. It is worth noting, however, that in Y = NO_2_ heptafulvenes, the X = NH_2_ group only in the γ position increases the aromaticity—the HOMA, EDDB, and FLU values for NO_2_–NH_2_-β indicate a lower aromaticity than that in monosubstituted NO_2_–H heptafulvene. So, the π-electron delocalization between substituents in the γ derivatives increases the cyclic delocalization and thus the aromaticity of the heptafulvene ring, contrary to the less aromatic rings in the β systems. This is caused by the fact that the resonance interaction between Y = NO_2_ and X = NH_2_ in the γ position occurs along the longer delocalization path (Figure 2), which passes through almost the entire ring, and not just a small part of it as in the case of X in the β position. Consequently, the cyclic delocalization in the β systems encounters two obstacles in the form of short, highly localized double bonds (visible in the EDDB plot, Figure 2, and from the bond lengths, Table S6), whereas in γ, due to equalization of the bonds along the delocalization path, only one such bond within the ring is present. The isosurfaces of the EDDB_P_(π) function, which present only cyclically delocalized π electrons (Table S5), illustrate the beneficial effect of the γ-NH_2_ substitution on delocalization even more clearly. The effect of X or Y substitution can also be extracted in the form of differential EDDB maps presented in Table S4. Cis and trans isomers of disubstituted derivatives also show differences in the aromaticity and stability, the latter below 2 kcal/mol in all cases (Table S1). As a general rule, more stable isomers have a larger population of electron delocalized within the rings, as evidenced by EDDB_P_(π) (Table S1). Three exceptions to this case are the (p) NH_2_–NO_2_-β, NO_2_–NO_2_-γ, and NO_2_–NH_2_-γ systems, where the more stable isomer has less electrons delocalized within the ring, albeit in these cases the differences in aromaticity and stability are small (below 0.5 kcal/mol). Considering the β derivatives of both penta- and heptafulvenes, for X = NH_2_ the cis isomer is always more stable while for X = NO_2_ the trans isomer is always more stable. In the γ derivatives, the trans isomer is preferred when the X and Y groups are the same but the cis isomer is preferred when they are different.

**Figure 1:**
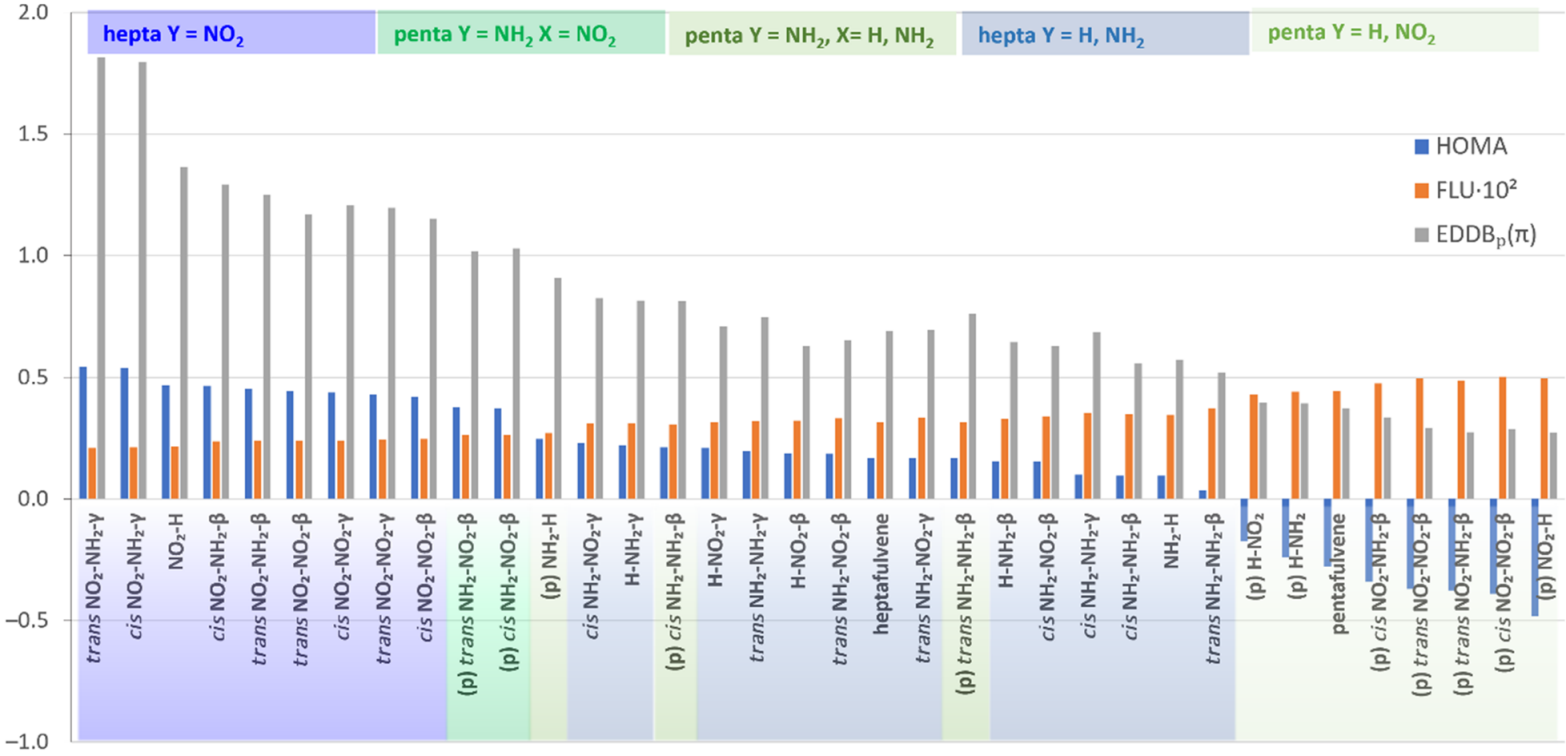
Values of HOMA, FLU aromaticity indices, and EDDB_P_(π) sorted by decreasing HOMA value; (p) pentafulvene derivatives.

**Figure 2:**
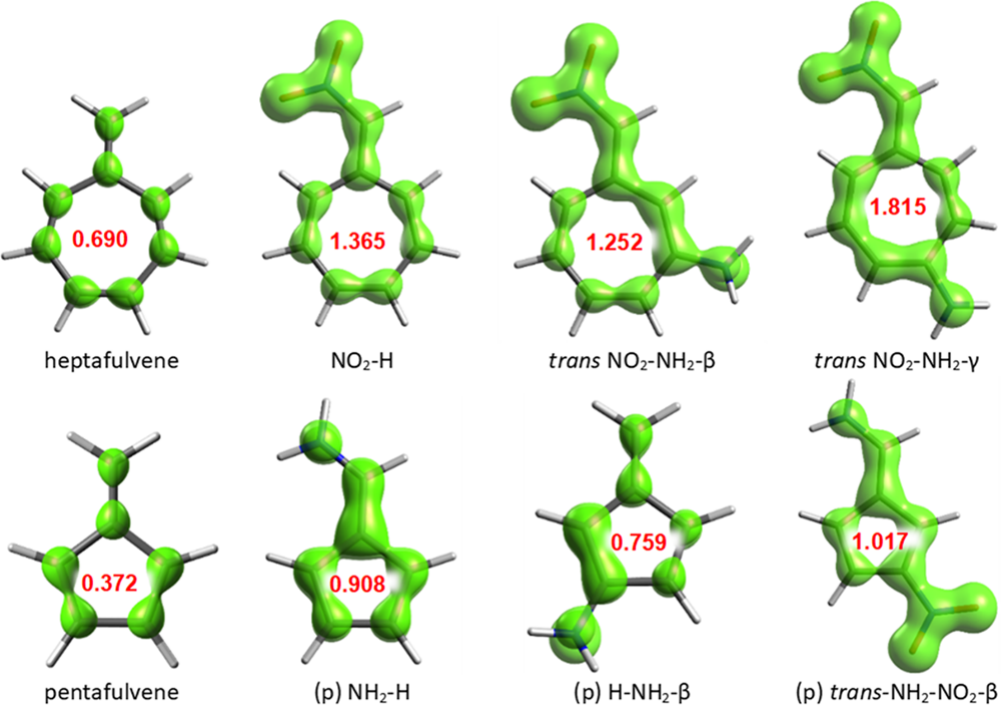
EDDB_H_ isosurfaces—visual representations of global electron delocalization (excluding H atoms), isovalue = 0.015; all systems are shown in Table S3. In red, the number of cyclically delocalized π electrons in ring is given, evaluated by EDDB_P_(π).

HOMA and FLU indices are linearly correlated with *R*^2^ = 0.988, whereas the EDDB_P_(π) vs HOMA relation can be approximated with an exponential function (*y* = *a* + *b*·exp(*c*·*x*), *R*^2^ = 0.976, see Figure S4). EDDB predicts higher aromaticity for (p) trans NH_2_–NH_2_-β and cis NH_2_–NH_2_-γ systems than HOMA, but the general trend of aromaticity change is maintained. The HOMA values and electronic properties of the Y substituents, described by cSAR(Y), are well correlated (Figure 3a). Additionally, the trendlines for penta- and heptafulvene series have opposite slopes. This means that in pentafulvenes, the aromaticity increases with the electron-donating and in heptafulvenes with the electron-withdrawing properties of Y. The properties of Y can be modified by substituents in the ring—the X group with opposite electronic properties increases the electron-donating and -withdrawing power of Y = NH_2_ and NO_2_, respectively. It is also worth noting that the variability of HOMA in pentafulvenes is larger than in that heptafulvenes, as evidenced by the more than two times higher absolute value of the slope in the pentafulvene series. This results from the significant antiaromaticity of Y = NO_2_ pentafulvenes (large, negative values of HOMA). The electron-donating and -withdrawing properties of exocyclic =CH–Y fragments and X groups were quantitatively described by the charge of the substituent active region (cSAR) parameter.^25^ In Figure 3b, two linearly correlated series of points can be distinguished, corresponding to derivatives with groups X = NH_2_ (positive cSAR(X) values) and X = NO_2_ (negative values).

**Figure 3:**
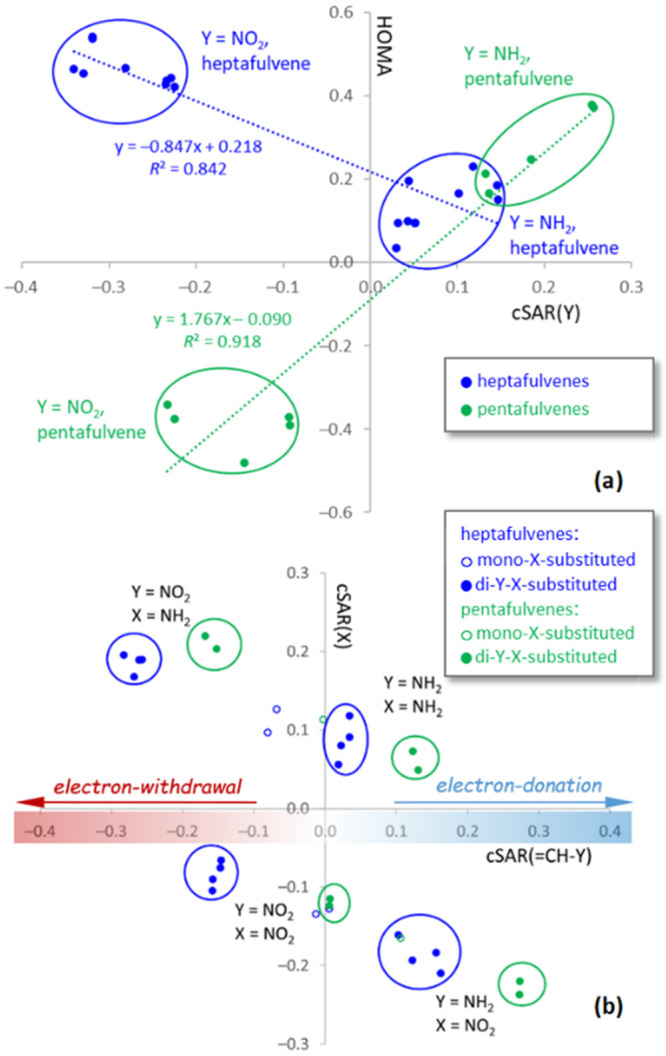
(a) Values of HOMA plotted against cSAR (Y) and (b) cSAR (X) plotted against cSAR (=CH–Y); interpretation of the cSAR values is given in b. Groups of points with specific Y and X groups are marked.

The correlation comes from the fact that the absolute values of cSAR(X) are higher in systems with Y and X groups with opposite electronic properties. Importantly, the values for pentafulvenes are always to the right of the values for heptafulvenes. This indicates that in pentafulvenes the =CH–Y group is always more electron donating (for Y = NH_2_) or less electron withdrawing (for Y = NO_2_) than in heptafulvenes. It appears that the π-electron deficiency in the pentafulvene ring promotes the electron donation by the =CH–Y group, while their excess in the heptafulvene ring promotes it being electron withdrawing. This is also observed in unsubstituted fulvenes (Y = H), where the =CH_2_ group is slightly electron donating in pentafulvene, cSAR(=CH–Y) = +0.047, and electron withdrawing in heptafulvene, cSAR(=CH–Y) = −0.055. In this way, fulvene systems can gain additional stability due to an increase in their aromatic character.

In the previous study of the SE in mono exo-substituted fulvenes,^16^ the pentafulvene ring was described as a highly electron-withdrawing reaction site whereas the heptafulvene was described as a highly electron-donating one, suggesting the use of σ_p_^+^ and σ_p_^–^ substituent constants, respectively. It appears that by adding the X substituent to the ring, the effective electron-donating or -withdrawing character by the ring–X system (R–X) increases further. This substitution alone has a small effect on R, but it has a substantial effect on the interaction of the entire R–X with the exocyclic Y group.

Comparing the β and γ substitution in heptafulvene derivatives, better transmission of the substituent effect between the Y and the X groups occurs in the β derivatives. This is evidenced by the differences in the absolute cSAR(Y) values for the β and γ systems (Figure S5). The differences are positive for systems with X and Y of opposite electronic properties, which indicates stronger interaction and larger charge transfer between groups for β than γ. In turn, when Y and X are the same, the clash between the two electron-donating/withdrawing groups tends to weaken their characteristic properties, so for the stronger interaction in β, the differences are negative (Figure S5). The above is also confirmed by the average values of the substituent effect stabilization energy (SESE, Table S1). The reason for the better transmission of the SE between Y and X in the β position is that the π conjugation pathway between Y and X is shorter for β (3 bonds) than that for γ (5 bonds). EDDB_H_ isosurfaces (Figure 2) confirm that in γ the longer, 5-bond pathway is indeed preferred.

Enhancement of the characteristic properties of the Y substituents is associated with a shortening of the C–Y bond. In Figure S6a, two linearly correlated series of points for Y = NH_2_ and NO_2_ can be noticed. Similarly, C–X bond lengths are well correlated with cSAR(X) (Figure S7). In the latter case, however, the points for penta- and heptafulvene derivatives are separated—the C–X bond lengths in pentafulvenes are shorter by about 0.04 Å for X = NO_2_ and 0.02 Å for X = NH_2_ than those in the corresponding heptafulvenes due to the weaker steric effects within the 5-membered ring. In Figure S6b, the electronic properties of the =CH–Y exo group are linearly correlated with the exo C=C bond length. In this case, the length of the C=C bond increases with the electron-donating or -withdrawing strength of =CH–Y. Two V-shaped series of points correspond to the penta- and heptafulvenes—the lengths of the C=C bonds in pentafulvenes are shorter. In Y = NH_2_ pentafulvene derivatives, the C=C bonds are longer than those for Y = NO_2_; in heptafulvene the opposite is observed. This is associated with the fact that the NH_2_ group is strongly electron donating in pentafulvenes; this strong conjugation between the NH_2_ and the ring causes the C–N bond to be shortened and the exo C=C bond to be stretched. In heptafulvenes, the same occurs in the Y = NO_2_ derivatives. Accordingly, the C=C bond lengths are well correlated (Figure S8) with the aromaticity described by HOMA—the longer the bond and stronger the conjugation, the higher the aromaticity.

## Computational Methods

Quantum chemical DFT calculations were performed in the Gaussian 16 program, rev. A.01,^26^ using the B3LYP functional with 6-311++G(d,p).^27^ Vibrational frequencies were calculated afterward to confirm that the optimized geometries correspond to the minima on the potential energy surface. Two conformations have been checked in the diamine derivatives. The first one corresponds to the two amino groups being rotated so that their lone pairs are facing the same direction, whereas in the second one the groups are rotated by 180° relative to each other—their lone pairs face opposite directions. The lower energy conformers were considered in further analyses.

π-Electron delocalization was assessed by three methods: harmonic oscillator model of aromaticity (HOMA),^28^ electron fluctuation index (FLU)^29^ and electron density of delocalized bonds (EDDB).^22^ EDDB_H_ evaluates global electron delocalization, while EDDB_P_(π) counts only cyclically delocalized π electrons in the ring. In addition, ASE^30^ and NICS^24^ were also computed; details are provided in the Supporting Information. The electronic properties of the substituents were evaluated quantitatively using the charge of the substituent active region (cSAR) parameter.^25^

## Data Availability

The data underlying this study are available in the published article and its Supporting Information.

## Supporting Information Available

Computational details, supplementary tables, and figures (PDF)
Cartesian coordinates (.xyz files) with B3LYP/6-311++G(d,p)-optimized geometries (ZIP)
