# Supplementary material for: Substituent Effect versus Aromaticity—A Curious Case of Fulvene Derivatives
Source: J Org Chem. 2023 Sep 29;88(20):14775–80. doi: 10.1021/acs.joc.3c01539 (PMC10594647; doi:10.1021/acs.joc.3c01539)
Supplement: Supplementary file 1 — jo3c01539_si_001.pdf [file jo3c01539_si_001.pdf]

Supporting Information

Substituent effect versus aromaticity – a curious case of fulvene derivatives

Pawel A. Wieczorkiewicz[a,*], Krzysztof K. Zborowski[b], Tadeusz M. Krygowski[c], Halina Szatylowicz[a,*]

[a] Faculty of Chemistry, Warsaw University of Technology, Noakowskiego 3, Warsaw, 00-664, Poland,[b] Faculty of Chemistry, Jagiellonian University in Kraków, Gronostajowa 2, Kraków, 30-387, Poland,[c] Department of Chemistry, University of Warsaw, Pasteura 1, Warsaw, 02-093, Poland

* correspondence: pawel.wieczorkiewicz.dokt@pw.edu.pl (P.A.W.), halina.szatylowicz@pw.edu.pl (H.S.)

Table of Contents

Computational Methods **S2**

Figure S1. Structures of all studied systems along with the notation used in the manuscript. S2

Figure S2. Definition of cSAR and interpretation of its value. q_X is the sum of atomic charges of all atoms forming a substituent X, while q_{ipso} is the atomic charge at the *ipso* atom. S5

Supplementary Tables and Figures **S5**

Figure S3. Calculated values of cSAR(=CH-Y) and cSAR(X), sorted by (a) increasing cSAR(=CH-Y) and (b) by increasing cSAR(X). S5

Table S1. Relative stability of hepta- and pentafulvene derivatives and calculated values of aromaticity indices for fulvene ring. S6

Table S2. Collected data generated in this study. Electronic properties of groups X, Y and exocyclic fragment =CH-Y, evaluated by cSAR. Values of aromaticity indices, lengths of C-X (d_{CX}) C-Y (d_{CY}) and exocyclic C=C ($d_{C=C}$) bonds (in Å), electron density at C=C bond critical point (ρ_{BCP}, in e·B^{-3}), corresponding laplacian of electron density ($\nabla^2\rho_{BCP}$, in e·B^{-5}) and C=C bond ellipticity (ε).. .... S8

Figure S4. Dependences between the calculated values of aromaticity indices. S10

Figure S5. Differences in absolute values of cSAR(Y) for the β and γ heptafulvene derivatives. ... S10

Figure S6. Lengths of C-Y bonds (d_{CY}) and C=C bonds ($d_{C=C}$) plotted against the values of (a) cSAR(Y) and (b) cSAR(=CH-Y), respectively. S11

Figure S7. Dependences between (a) CX bond length and cSAR(X), (b) CY bond length and cSAR(Y). S11

Figure S8. Dependences between HOMA of the hepta- and pentafulvene ring and C=C bond length. S12

Figure S9. Dependence between exocyclic C=C bond parameters – its length and electron density at bond critical point, ρ_{BCP}. S12

Figure S10. Correlations between HOMA and (a) ASE or (b) NICS(1)$_{zz}$ aromaticity indices. S12

Table S3. Isosurfaces of the EDDB$_H$ function (isovalue=0.015), representing the global electron delocalization in hepta- and pentafulvene derivatives. S13

Table S4. Differential EDDB maps.. .... S17

Table S5. Isosurfaces of the EDDB$_P$(π) function (isovalue=0.005), representing the π-electron cyclic delocalization in hepta- and pentafulvene derivatives. S18

Table S6. Bond lengths (in Å) in studied penta- and heptafulvene derivatives. B3LYP/6-311++G(d,p) results. S23

References **S33**

Computational Methods

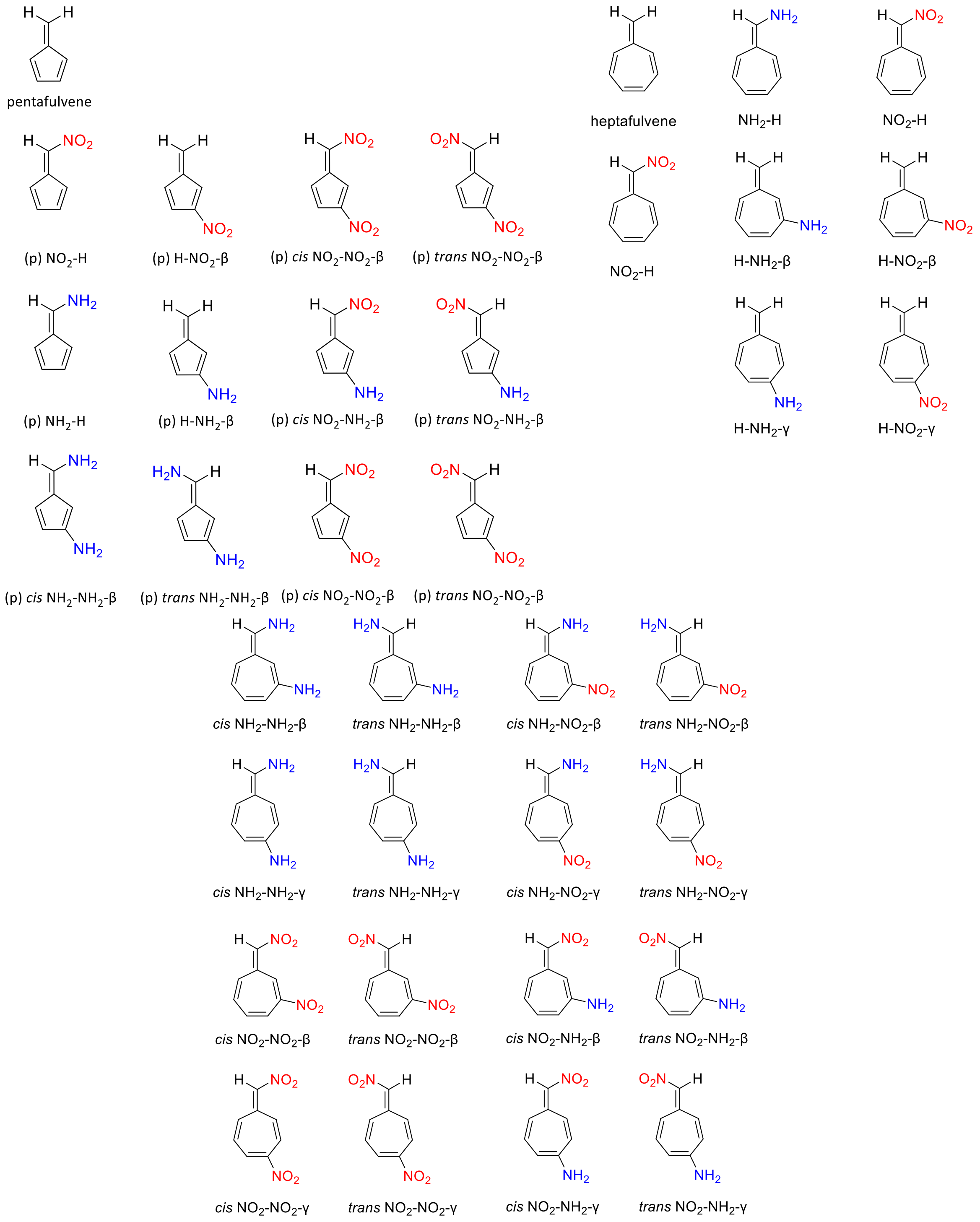

Figure S1. Structures of all studied systems along with the notation used in the manuscript.

Quantum chemical DFT calculations were performed in the Gaussian 16 program rev. A.01[1] using B3LYP functional with 6-311++G(d,p).[2] Vibrational frequencies were calculated afterwards, to confirm that the optimized geometries correspond to the minima on the potential energy surface. Two conformations have been checked in the diamine derivatives; first one corresponding to the two amino

groups rotated so that their lone pairs are facing the same direction, whereas in the second one the groups are rotated by 180 degrees relative to each other – their lone pairs face opposite directions. The lower energy conformers were considered in further analyses.

π-Electron delocalization was assessed using several methods: Harmonic Oscillator Model of Aromaticity (HOMA), Electron Fluctuation Index (FLU), Aromatic Stabilization Energy (ASE), Nuclear Independent Chemical Shift (NICS), and Electron Density of Delocalized Bonds (EDDB).

HOMA [3,4] is a geometry-based aromaticity index, which can be calculated from Equation 1:

$$\mathrm{HOMA} = 1 - \frac{1}{n}\sum_{i}^{n} \alpha_{\mathrm{j}}(d_{\mathrm{opt,j}} - d_{\mathrm{j,i}})^2, \tag{1}$$

where n is the number of bonds taken into account when carrying out the summation, i indicates the type of bond, α_{j} is an empirical normalization constant (for CC bond, α_{CC} = 257.7), $d_{\mathrm{opt,j}}$ is the optimal length of a given bond assumed to be realized in fully aromatic systems with HOMA = 1 (for CC bond, $d_{\mathrm{opt,CC}}$ = 1.388 Å), and $d_{\mathrm{j,i}}$ is an actual bond length in the studied system.

FLU index is an index derived from the electronic structure of the molecule, namely the values of electron sharing indices (ESI) between pairs of atoms.[5] FLU can be calculated from Equation 2, in that case the ESI used are delocalization indices between two atoms, A and B, δ(A, B). δ_{ref}(A, B) is a reference value of δ(A, B); for C-C bonds it corresponds to the value for the bond in benzene.

$$FLU(\mathcal{A}) = \frac{1}{N}\sum_{i=1}^{N}\left[\left(\frac{V(A_i)}{V(A_{i-1})}\right)^{\alpha}\left(\frac{\delta(A_i, A_{i-1}) - \delta_{\mathrm{ref}}(A_i, A_{i-1})}{\delta_{\mathrm{ref}}(A_i, A_{i-1})}\right)\right]^2 \tag{2}$$

The ring considered in Equation 2 is formed by atoms in the string $\{\mathcal{A}\} = \{A_1, A_2, \ldots A_N\}$, $A_0 \equiv A_N$ and the atomic delocalization $V(A)$ is defined by Equation 3:

$$V(A) = \sum_{A \neq B} \delta(A, B) \tag{3}$$

while α is a function that ensures that the ratio of atomic delocalizations in Eq. 2 is always greater or equal to 1 (Equation 4):

$$\alpha = \begin{cases} 1 & V(A_i) > V(A_{i-1}) \\ -1 & V(A_i) \leq V(A_{i-1}) \end{cases} \tag{4}$$

The ESI used can be the delocalization index, fuzzy bond orders, or Mayer-Wiberg bond orders.[6] In our particular case, FLU was calculated from fuzzy bond orders using scripts implemented in Multiwfn program.[7] It should be emphasized that the FLU (Equation 2) is close to 0 in aromatic systems and increases as the molecule moves away from the aromatic reference.

EDDB is a recent method which has its roots in the orbital communication theory. It uses the natural orbital representation (NAO) of the wavefunction and several transformations to decompose the total electron density into electrons which are localized on single atoms – lone pairs and core electrons (Electron Density Localized on Atoms, EDLA), electrons localized between atomic pairs (Electron Density of Localized Bonds, EDLB), and electrons delocalized between several bonds (EDDB). The atomic populations or density of delocalized electrons can be further analyzed in order to describe delocalization quantitatively or visualize graphically. Full theory behind the EDDB method can be found in papers by Szczepanik et al.[8,9] Various variants of EDDB exist. $\mathrm{EDDB_G}$ (G for global) evaluates the global electron delocalization, $\mathrm{EDDB_H}$ evaluates the global electron delocalization without contribution from H atoms, $\mathrm{EDDB_F}$ (F for fragment) evaluates delocalization in selected molecular fragment, $\mathrm{EDDB_P}$ (P for

pathway) evaluates electron delocalization for a particular cyclic delocalization pathway. In each variant contributions from electrons at σ, π, δ and ϕ orbitals can be dissected, for example $EDDB_P(\pi)$ evaluates the cyclic π-electron delocalization associated with aromaticity.

ASE is an index that goes to the very roots of aromaticity. From the beginning, high stability of benzene was observed, and later of other aromatic compounds. Thus, the idea of estimating the ASE and making it a measure of aromaticity arose. This energy used to be estimated on the basis of experimental data, nowadays on the basis of quantum-chemical calculations of the energy difference of various chemical reactions between aromatic and non-aromatic compounds.[10] Unfortunately, such energy estimation is not an easy and simple task. Plenty types of chemical reactions have been proposed so far. In this work, we used probably the most efficient method for estimating ASE (only two chemical compounds are involved in the reaction), the isomerization method.[11] This is based on the energy difference between the methyl derivative of the aromatic system and its nonaromatic exocyclic methylene isomer. Unfortunately, the estimation of the aromatic stabilization energy by the isomerization method (like other methods of calculating ASE) is disturbed by many factors, such as changes in the ring stress energy (obviously not the same for the aromatic and non-aromatic derivative) or the position in the ring where the exocyclic methyl and methylene groups are located. Also, the compounds studied in this work can potentially be modified for the isomerization reactions at different places. Therefore, for the purposes of this work, the exocyclic group was always substituted in the beta position (on the opposite side of the ring from the amine or nitro groups), and the CH_2 group appearing after isomerization in the ring in the adjacent alpha position.

NICS is a magnetic index of aromaticity. It was originally defined as the absolute magnetic shielding at the geometrical center of an investigated ring[12] and has had many modifications.[13] One of these modifications, $NICS(1)_{zz}$ (where the point at which we calculate the chemical shift is one angstrom above the center of the ring, and only the component of the magnetic tensor perpendicular to the plane of the ring is taken into account), is used here.[14] The calculation were carried out using the GIAO method at the B3LYP/6-311++G(d,p) computational level.[15] In the case of significantly bend rings (to avoid interference due to the proximity of atomic nuclei), the NICS value was calculated on the side of the ring where the chemical shift calculation point is positioned further away from the atoms of a studied molecule.

Electronic properties of substituents were evaluated quantitatively using the charge of the substituent active region (cSAR) parameter.[16] Its definition is presented in Figure S2. Positive cSAR values indicate the deficit of electrons in the substituent active region, *i.e.* the substituent is electron-donating. Negative values represent an excess of electrons in the active region of the substituent, indicating its electron-withdrawing properties. To allow comparison with our other results (e.g. data on benzene derivatives, presented in some figures in the Supplementary Materials), the atomic charges used to calculate cSAR were obtained by the Hirshfeld method.[17] cSAR was calculated for substituents X and Y, cSAR(X) and cSAR(Y), as well as for the entire exocyclic fragment =CH-Y, cSAR(=C-Y). Substituent Effect Stabilization Energy (SESE) is the energetic substituent effect descriptor. It evaluates energy associated with interaction between two substituents and is calculated as the energy difference of reactions of type: Y-R-X + R => R-X + R-Y. AIM analysis was performed in Multiwfn program.[7]

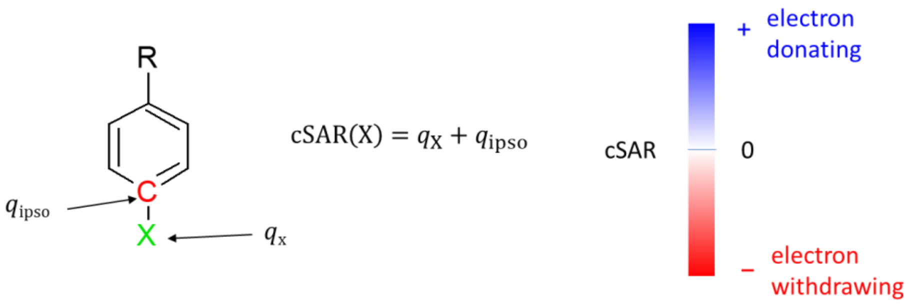

Figure S2. Definition of cSAR and interpretation of its value. q_X is the sum of atomic charges of all atoms forming a substituent X, while q_{ipso} is the atomic charge at the *ipso* atom.

Supplementary Tables and Figures

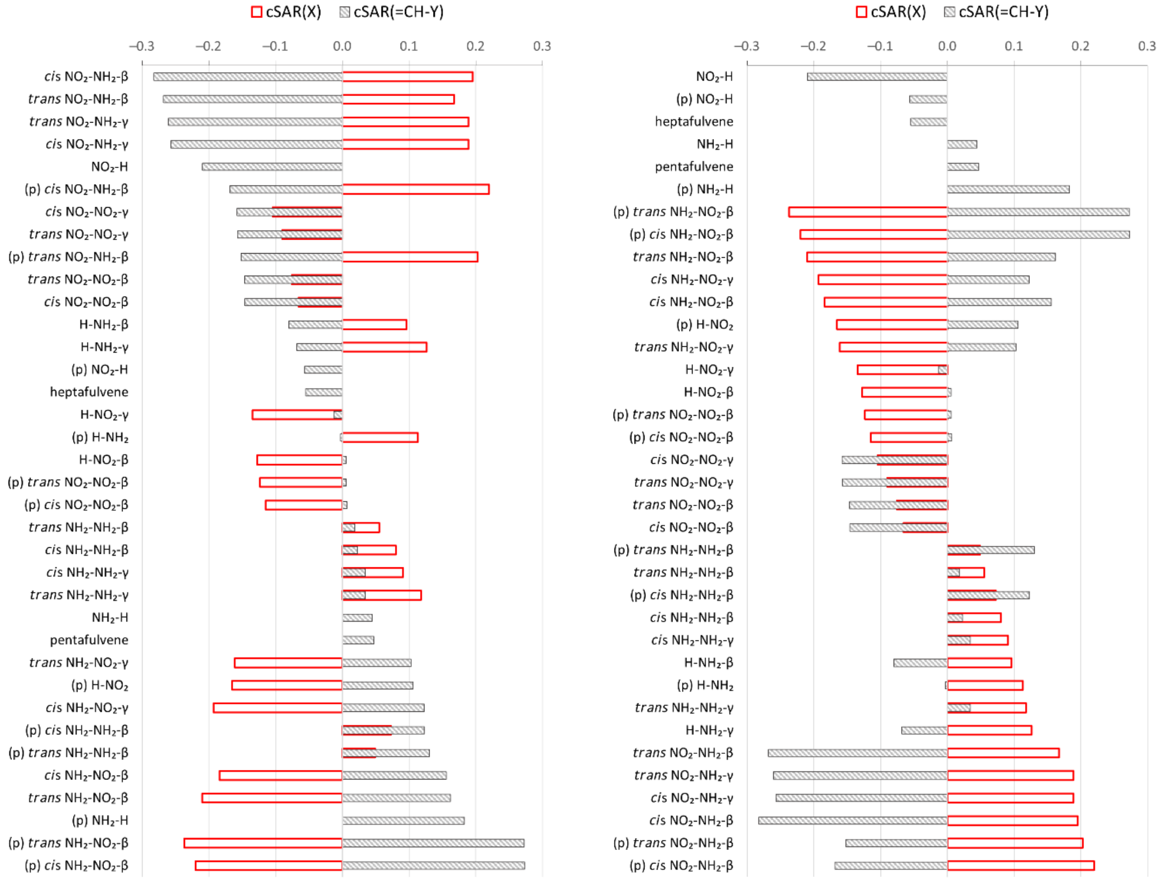

(a) (b)

Figure S3. Calculated values of cSAR(=CH-Y) and cSAR(X), sorted by (a) increasing cSAR(=CH-Y) and (b) by increasing cSAR(X).

Table S1. Relative stability of hepta- and pentafulvene derivatives and calculated values of aromaticity indices for fulvene ring. ΔE(*cis*,*trans*) compares the stability of *cis* and *trans* diastereoisomers of given compound; ΔE compares the stability of all configurational isomers with the same chemical formula (each group is separated by double borders). FLU*/FLU is the value of FLU for unsubstituted hepta- or pentafulvene divided by the value for given system.

heptafulvenes

	ΔE(*cis*,*trans*) /kcal·mol^{-1}	ΔE /kcal·mol^{-1}	SESE /kcal·mol^{-1}	EDDB$_P$(π) (7)	HOMA(7)	FLU*/FLU	FLU
heptafulvene				0.690	0.167	1.00	0.032
H-NH$_2$-β		1.77		0.645	0.154	0.96	0.033
H-NH$_2$-γ		0.00		0.816	0.221	1.01	0.031
NH$_2$-H		2.07		0.572	0.095	0.92	0.035
H-NO$_2$-β		3.82		0.629	0.188	0.98	0.032
H-NO$_2$-γ		3.32		0.707	0.208	1.01	0.031
NO$_2$-H		0.00		1.365	0.466	1.47	0.022
cis NO$_2$-NH$_2$-β	0.00	0.00	-4.35	1.292	0.465	1.34	0.0236
trans NO$_2$-NH$_2$-β	1.98	1.98	-2.37	1.252	0.453	1.33	0.0238
cis NO$_2$-NH$_2$-γ	0.00	0.31	-2.28	1.795	0.537	1.49	0.0212
trans NO$_2$-NH$_2$-γ	0.03	0.34	-2.24	1.815	0.543	1.51	0.0209
cis NH$_2$-NO$_2$-β	1.35	5.29	-3.19	0.627	0.152	0.93	0.0339
trans NH$_2$-NO$_2$-β	0.00	3.93	-4.54	0.653	0.185	0.96	0.0330
cis NH$_2$-NO$_2$-γ	0.00	4.79	-3.18	0.825	0.230	1.02	0.0310
trans NH$_2$-NO$_2$-γ	1.41	6.20	-1.78	0.694	0.166	0.95	0.0333
cis NO$_2$-NO$_2$-β	0.97	1.83	5.25	1.153	0.421	1.29	0.0246
trans NO$_2$-NO$_2$-β	0.00	0.87	4.28	1.171	0.443	1.33	0.0237
cis NO$_2$-NO$_2$-γ	0.23	0.23	4.15	1.206	0.436	1.32	0.0240
trans NO$_2$-NO$_2$-γ	0.00	0.00	3.92	1.196	0.428	1.30	0.0243
cis NH$_2$-NH$_2$-β	0.00	1.73	-0.27	0.556	0.096	0.91	0.0349
trans NH$_2$-NH$_2$-β	1.91	3.64	1.64	0.520	0.036	0.85	0.0373
cis NH$_2$-NH$_2$-γ	1.21	1.21	0.98	0.686	0.100	0.89	0.0354
trans NH$_2$-NH$_2$-γ	0.00	0.00	-0.23	0.746	0.197	0.99	0.0320

pentafulvenes

	ΔE(*cis*,*trans*) /kcal·mol^{-1}	ΔE /kcal·mol^{-1}	SESE /kcal·mol^{-1}	EDDB$_P$(π) (5)	HOMA(5)	FLU*/FLU	FLU
pentafulvene				0.372	-0.278	1.00	0.0443
(p) H-NO$_2$		0.00		0.397	-0.174	1.03	0.0429
(p) NO$_2$-H		3.48		0.272	-0.482	0.89	0.0496
(p) H-NH$_2$		4.67		0.393	-0.240	1.01	0.0440

(p) NH_2-H		0.00		0.908	0.247	1.64	0.0270
(p) *cis* NO_2-NO_2-β	0.34		4.08	0.288	-0.390	0.88	0.0502
(p) *trans* NO_2-NO_2-β	0.00		3.74	0.290	-0.372	0.90	0.0494
(p) *cis* NH_2-NH_2-β	0.00		2.27	0.813	0.212	1.45	0.0305
(p) *trans* NH_2-NH_2-β	1.70		3.97	0.759	0.166	1.40	0.0316
(p) *cis* NO_2-NH_2-β	0.00	7.50	-5.34	0.336	-0.341	0.94	0.0473
(p) *trans* NO_2-NH_2-β	1.36	8.86	-3.98	0.274	-0.376	0.91	0.0486
(p) *cis* NH_2-NO_2-β	0.49	0.49	-4.21	1.029	0.372	1.68	0.0264
(p) *trans* NH_2-NO_2-β	0.00	0.00	-4.69	1.017	0.378	1.68	0.0264

Table S2. Collected data generated in this study. Electronic properties of groups X, Y and exocyclic fragment =CH-Y, evaluated by cSAR. Values of aromaticity indices, lengths of C-X (d_{CX}) C-Y (d_{CY}) and exocyclic C=C ($d_{C=C}$) bonds (in Å), electron density at C=C bond critical point (ρ_{BCP}, in e·B^{-3}), corresponding laplacian of electron density ($\nabla^2\rho_{BCP}$, in e·B^{-5}) and C=C bond ellipticity (ε). Planarity of fulvene rings in studied compounds is color coded in the first column: green – planar, orange – bent. Systems are sorted by decreasing HOMA value.

	cSAR(X)	cSAR(Y)	cSAR(=CH-Y)	EDDB$_P$(π)	HOMA	FLU	NICS(1)$_{zz}$ /ppm	ASE /kcal·mol^{-1}	d_{CX}	d_{CY}	$d_{C=C}$	ρ_{BCP}	$\nabla^2\rho_{BCP}$	ε
***trans* NO_2-NH_2-γ**	0.189	-0.320	-0.261	1.815	0.543	0.0209	13.98	1.815	1.3769	1.4167	1.3835	0.3130	-0.8639	0.3007
***cis* NO_2-NH_2-γ**	0.189	-0.320	-0.257	1.795	0.537	0.0212	13.13	1.795	1.3765	1.4169	1.3830	0.3132	-0.8643	0.3008
**NO_2-H**		-0.281	-0.210	1.365	0.466	0.0216	9.83	1.365		1.4236	1.3785	0.3160	-0.8777	0.3091
***cis* NO_2-NH_2-β**	0.196	-0.341	-0.282	1.292	0.465	0.0236	9.18	1.292	1.3781	1.4123	1.3879	0.3104	-0.8498	0.2993
***trans* NO_2-NH_2-β**	0.167	-0.329	-0.268	1.252	0.453	0.0238	9.04	1.252	1.3862	1.4165	1.3852	0.3119	-0.8568	0.3054
***trans* NO_2-NO_2-β**	-0.076	-0.229	-0.147	1.171	0.443	0.0237	10.32	1.171	1.5178	1.4315	1.3758	0.3181	-0.8898	0.3123
***cis* NO_2-NO_2-γ**	-0.105	-0.234	-0.158	1.206	0.436	0.0240	9.20	1.206	1.5042	1.4324	1.3731	0.3194	-0.8957	0.3145
***trans* NO_2-NO_2-γ**	-0.090	-0.235	-0.157	1.196	0.428	0.0243	9.22	1.196	1.5022	1.4324	1.3729	0.3195	-0.8958	0.3152
***cis* NO_2-NO_2-β**	-0.066	-0.225	-0.146	1.153	0.421	0.0246	6.59	1.153	1.5165	1.4319	1.3729	0.3196	-0.8966	0.3171
**(p) *trans* NH_2-NO_2-β**	-0.237	0.255	0.273	1.017	0.378	0.0264	8.39	1.017	1.4372	1.3447	1.3717	0.3196	-0.9236	0.2672
**(p) *cis* NH_2-NO_2-β**	-0.220	0.257	0.273	1.029	0.372	0.0264	10.24	1.029	1.4421	1.3448	1.3719	0.3192	-0.9213	0.2679
**(p) NH_2-H**		0.185	0.183	0.908	0.247	0.0270	8.16	0.908		1.3574	1.3637	0.3234	-0.9378	0.2893
***cis* NH_2-NO_2-γ**	-0.193	0.119	0.123	0.825	0.230	0.0310	4.34	0.825	1.4750	1.3747	1.3668	0.3228	-0.9099	0.3477
**H-NH_2-γ**	0.126		-0.068	0.816	0.221	0.0312	7.24	0.816	1.3941		1.3575	0.3278	-0.9380	0.3190
**(p) *cis* NH_2-NH_2-β**	0.073	0.133	0.123	0.813	0.212	0.0305	7.24	0.813	1.3926	1.3694	1.3607	0.3254	-0.9426	0.3039
**H-NO_2-γ**	-0.135		-0.013	0.707	0.208	0.0314	6.23	0.707	1.4935		1.3542	0.3306	-0.9541	0.3154
***trans* NH_2-NH_2-γ**	0.118	0.044	0.034	0.746	0.197	0.0320	4.91	0.746	1.3938	1.3912	1.3647	0.3235	-0.9092	0.3621
**H-NO_2-β**	-0.128		0.006	0.629	0.188	0.0322	11.59	0.629	1.5055		1.3566	0.3295	-0.9501	0.3094
***trans* NH_2-NO_2-β**	-0.210	0.146	0.162	0.653	0.185	0.0330	3.28	0.653	1.4761	1.3674	1.3736	0.3193	-0.8954	0.3304
**heptafulvene**			-0.055	0.690	0.167	0.0316	12.52	0.690			1.3553	0.3294	-0.9458	0.3222

	cSAR(X)	cSAR(Y)	cSAR(=CH-Y)	$EDDB_P(\pi)$	HOMA	FLU	$NICS(1)_{zz}$ /ppm	ASE /kcal·mol^{-1}	d_{CX}	d_{CY}	$d_{C=C}$	ρ_{BCP}	$\nabla^2\rho_{BCP}$	ε
***trans* NH_2-NO_2-γ**	-0.161	0.102	0.103	0.694	0.166	0.0333	3.69	0.694	1.4876	1.3808	1.3637	0.3242	-0.9139	0.3624
**(p) *trans* NH_2-NH_2-β**	0.049	0.137	0.131	0.759	0.166	0.0316	6.75	0.759	1.3995	1.3708	1.3605	0.3253	-0.9407	0.3083
**H-NH_2-β**	0.096		-0.080	0.645	0.154	0.0330	6.08	0.645	1.4052		1.3586	0.3270	-0.9317	0.3256
***cis* NH_2-NO_2-β**	-0.184	0.147	0.156	0.627	0.152	0.0339	2.28	0.627	1.4856	1.3680	1.3730	0.3190	-0.8902	0.3415
***cis* NH_2-NH_2-γ**	0.091	0.043	0.034	0.686	0.100	0.0354	3.90	0.686	1.4031	1.3954	1.3602	0.3260	-0.9205	0.3732
***cis* NH_2-NH_2-β**	0.080	0.032	0.023	0.556	0.096	0.0349	3.10	0.556	1.4074	1.3951	1.3613	0.3258	-0.9196	0.3711
**NH_2-H**		0.052	0.045	0.572	0.095	0.0345	3.06	0.572		1.3920	1.3605	0.3260	-0.9201	0.3734
***trans* NH_2-NH_2-β**	0.056	0.030	0.019	0.520	0.036	0.0373	3.62	0.520	1.4157	1.3993	1.3577	0.3273	-0.9245	0.3863
**(p) H-NO_2**	-0.165		0.106	0.397	-0.174	0.0429	3.32	0.397	1.4496		1.3434	0.3383	-1.0164	0.2683
**(p) H-NH_2**	0.113		-0.003	0.393	-0.240	0.0440	0.68	0.393	1.3815		1.3453	0.3356	-0.9952	0.2851
**pentafulvene**			0.047	0.372	-0.278	0.0443	1.20	0.372			1.3419	0.3432	-1.0099	0.2976
**(p) *cis* NO_2-NH_2-β**	0.220	-0.233	-0.169	0.336	-0.341	0.0473	-2.48	0.336	1.3541	1.4392	1.3551	0.3304	-0.9695	0.2898
**(p) *trans* NO_2-NO_2-β**	-0.124	-0.093	0.006	0.290	-0.372	0.0494	-1.14	0.290	1.4529	1.4643	1.3428	0.3388	-1.0176	0.2942
**(p) *trans* NO_2-NH_2-β**	0.203	-0.225	-0.152	0.274	-0.376	0.0486	-1.97	0.274	1.3577	1.4427	1.3536	0.3311	-0.9719	0.2929
**(p) *cis* NO_2-NO_2-β**	-0.115	-0.092	0.007	0.288	-0.390	0.0502	0.93	0.288	1.4555	1.4655	1.3421	0.3391	-1.0185	0.2952
**(p) NO_2-H**		-0.144	-0.057	0.272	-0.482	0.0496	-2.67	0.272		1.4602	1.3428	0.3383	-1.0138	0.2919

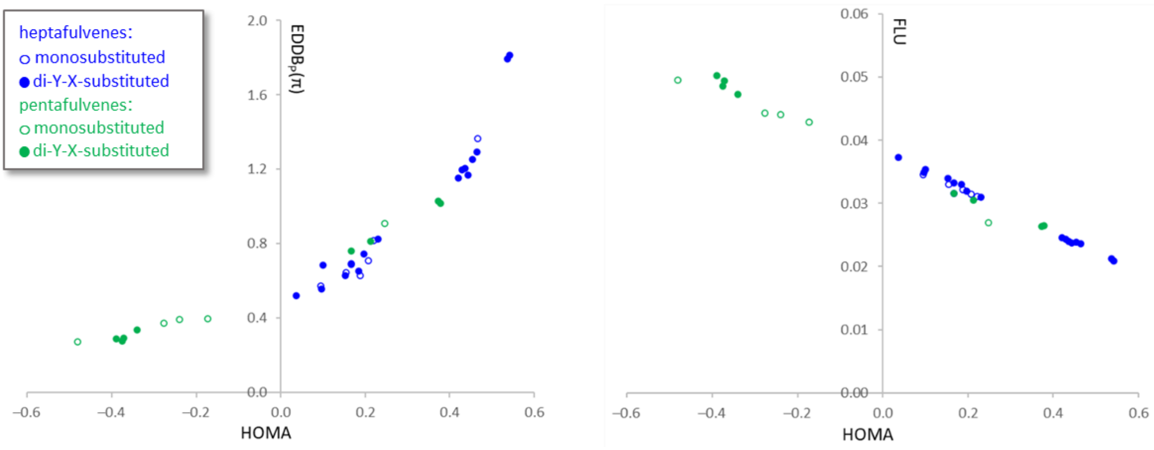

(a) (b)

Figure S4. Dependences between the calculated values of aromaticity indices. Fitting functions: (a) y = a+b·exp(c·x), R^2 = 0.976, a = 0.262 ± 0.036, b = 0.231 ± 0.033, c = 3.3627 ± 0.247; (b) y = a·x+b, R^2 = 0.989, a = -0.0300±0.0005, b=0.0373±0.0002.

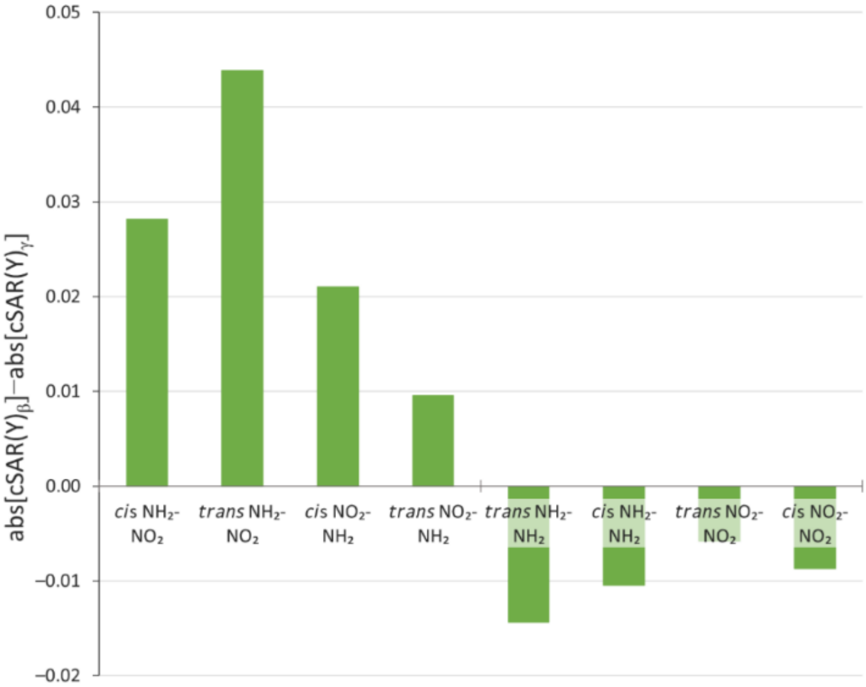

Figure S5. Differences in absolute values of cSAR(Y) for the β and γ heptafulvene derivatives.

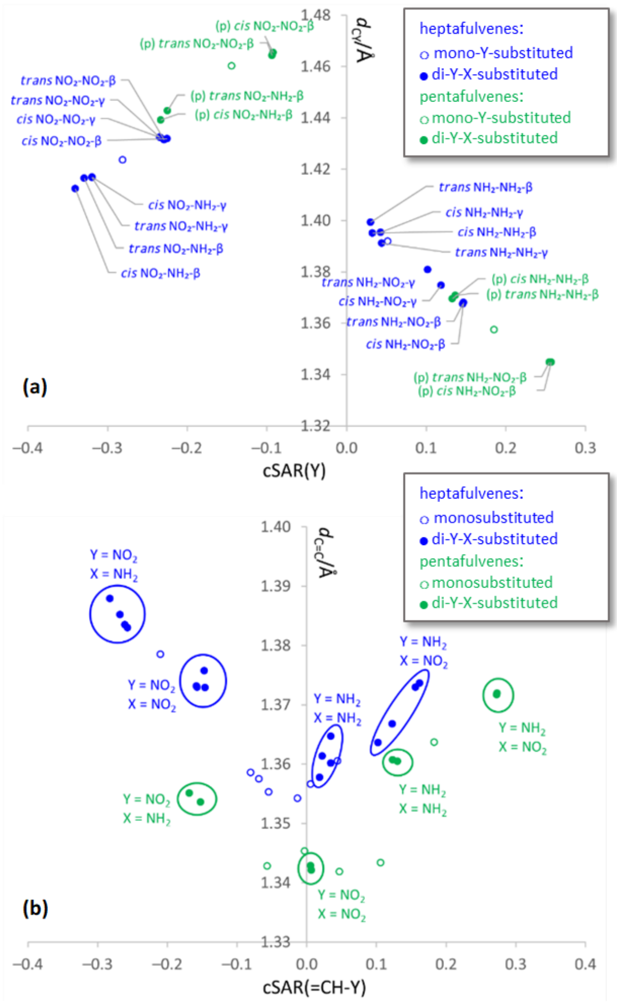

Figure S6. Lengths of C-Y bonds (d_{CY}) and C=C bonds ($d_{C=C}$) plotted against the values of (a) cSAR(Y) and (b) cSAR(=CH-Y), respectively.

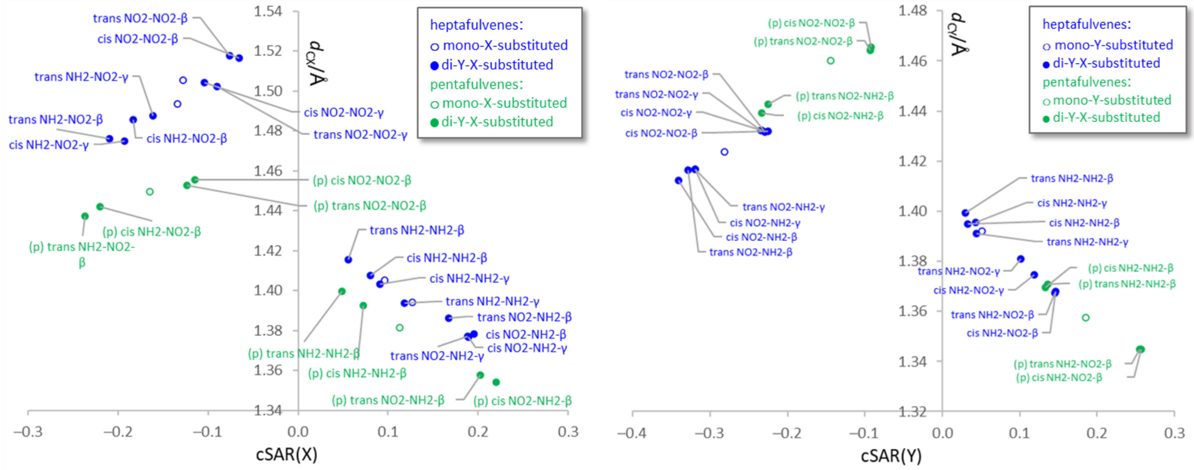

(a) (b)

Figure S7. Dependences between (a) CX bond length and cSAR(X), (b) CY bond length and cSAR(Y).

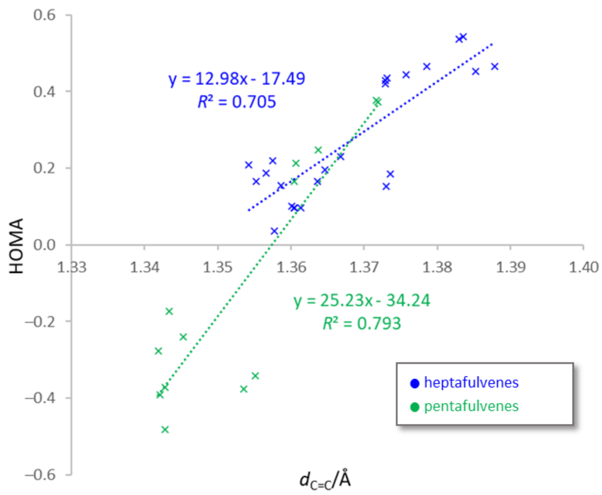

Figure S8. Dependences between HOMA of the hepta- and pentafulvene ring and C=C bond length.

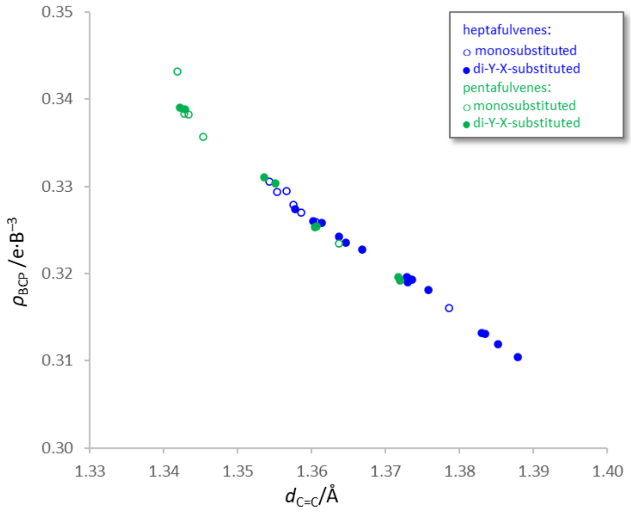

Figure S9. Dependence between exocyclic C=C bond parameters – its length and electron density at bond critical point, ρ_{BCP}.

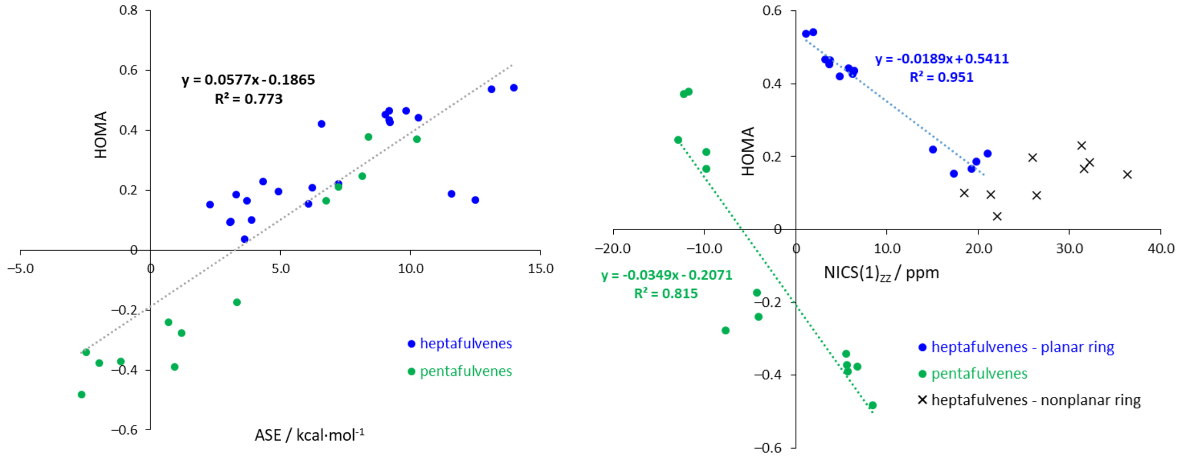

Figure S10. Correlations between HOMA and (a) ASE or (b) NICS(1)$_{zz}$ aromaticity indices.

Table S3. Isosurfaces of the $EDDB_H$ function (isovalue=0.015), representing the global electron delocalization in hepta- and pentafulvene derivatives.

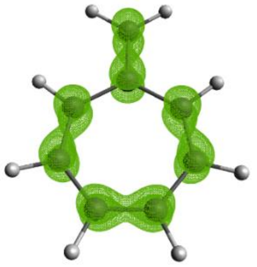

heptafulvene

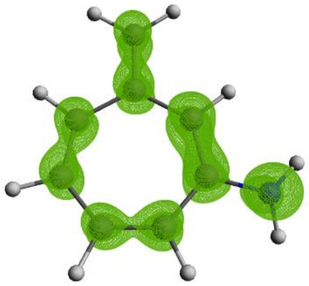

H-NH_2-β

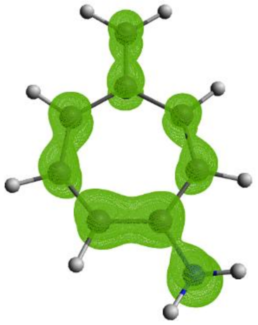

H-NH_2-γ

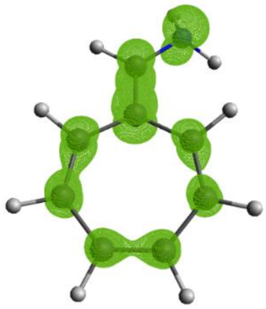

NH_2-H

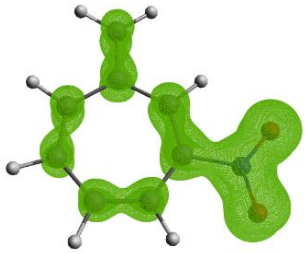

H-NO_2-β

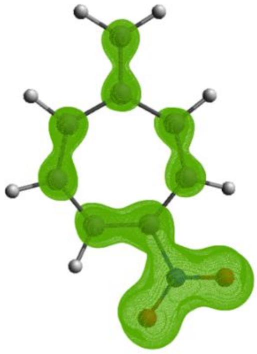

H-NO_2-γ

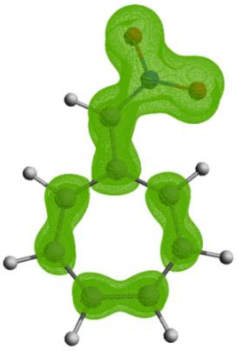

NO_2-H

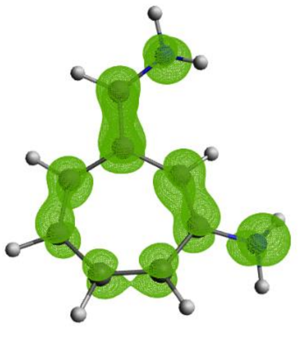

cis NH_2-NH_2-β

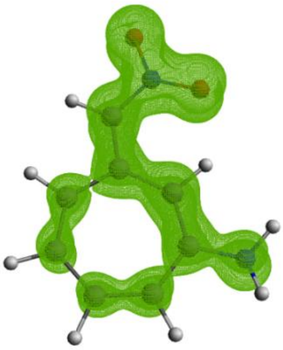

cis NO_2-NH_2-β

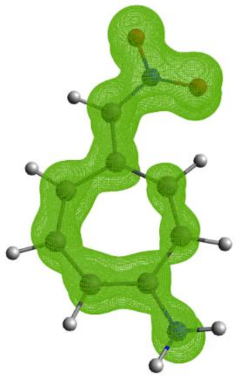

cis NO_2-NH_2-γ

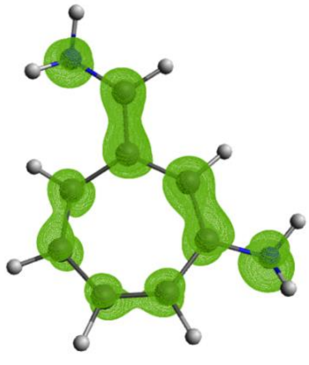

trans NH_2-NH_2-β

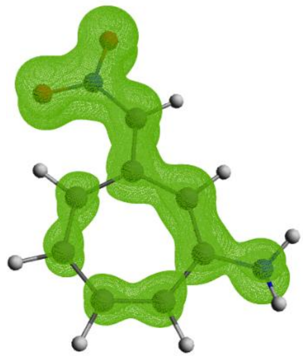

trans NO_2-NH_2-β

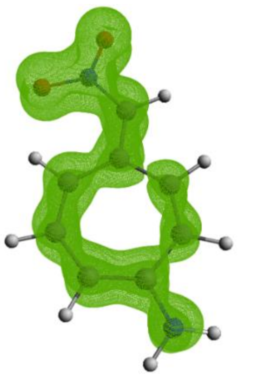

trans NO_2-NH_2-γ

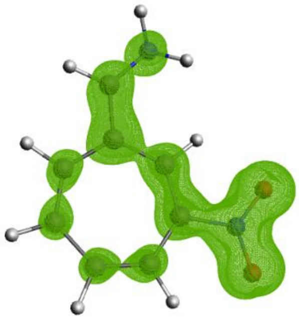

cis NH_2-NO_2-β

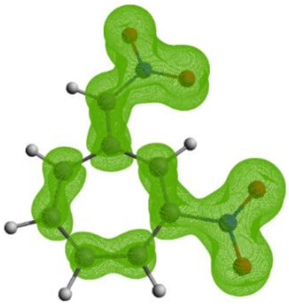

cis NO_2-NO_2-β

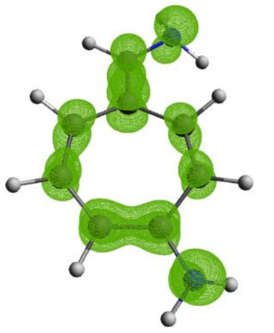

cis NH_2-NH_2-γ

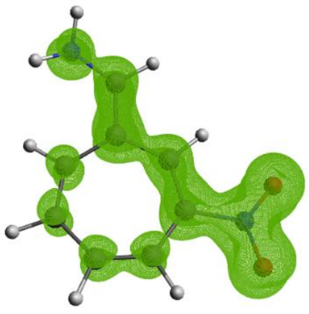

trans NH_2-NO_2-β

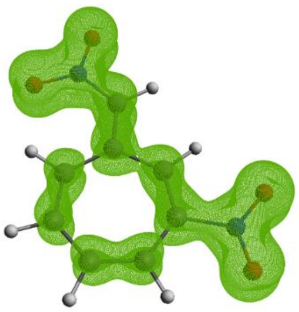

trans NO_2-NO_2-β

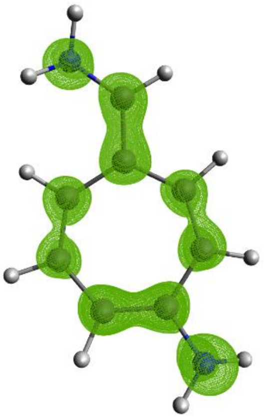

trans NH_2-NH_2-γ

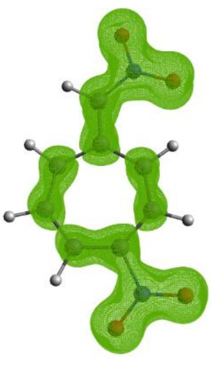

cis NO_2-NO_2-γ

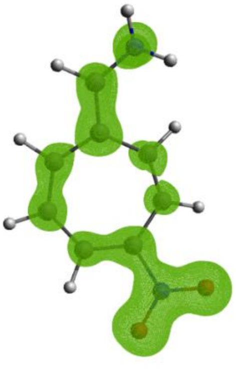

cis NH_2-NO_2-γ

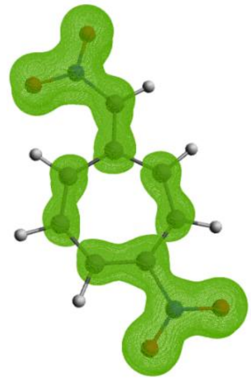

trans NO_2-NO_2-γ

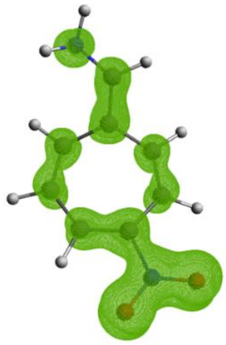

trans NH_2-NO_2-γ

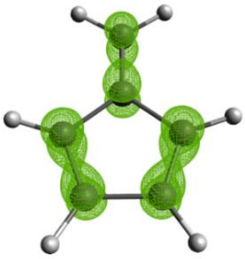

pentafulvene

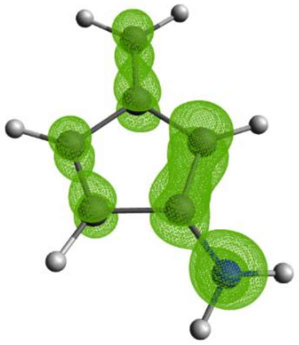

(p) H-NH_2-β

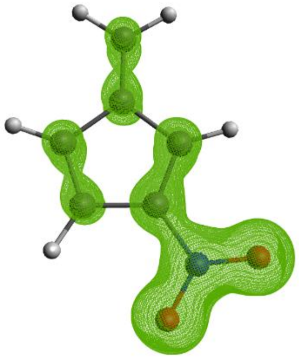

(p) H-NO_2-β

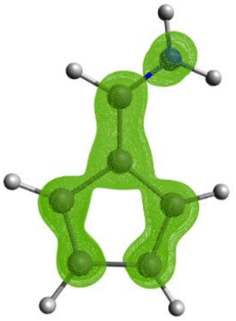

(p) NH_2-H

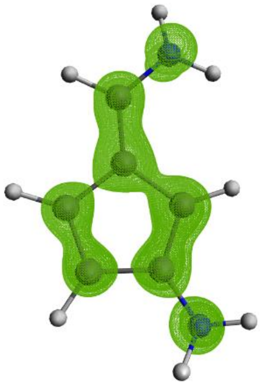

(p) cis NH_2-NH_2-β

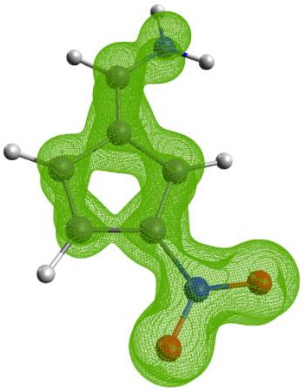

(p) cis NH_2-NO_2-β

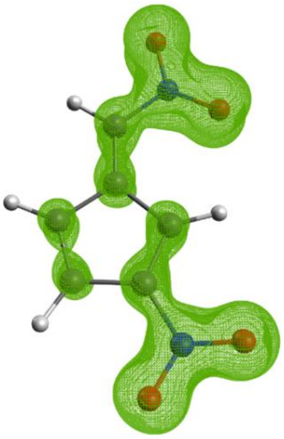

(p) cis NO_2-NO_2-β

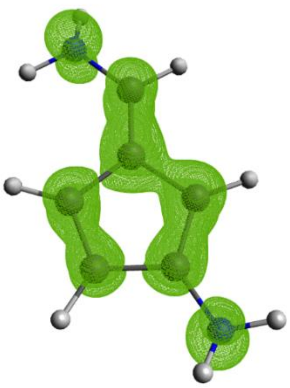

(p) trans NH_2-NH_2-β

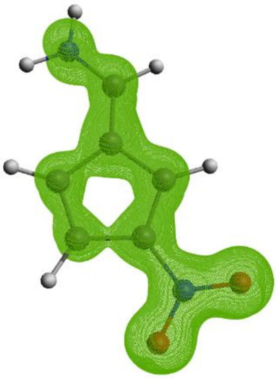

(p) trans NH_2-NO_2-β

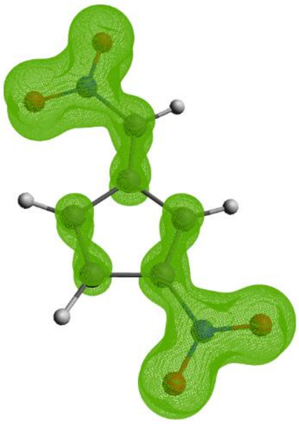

(p) trans NO_2-NO_2-β

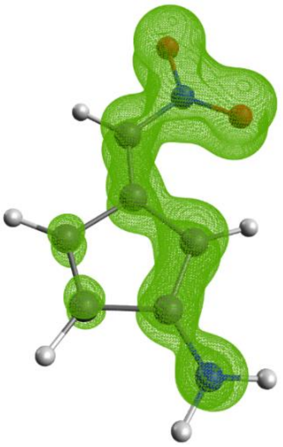

(p) cis NO_2-NH_2-β

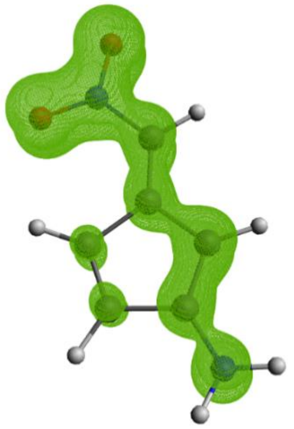

(p) trans NO_2-NH_2-β

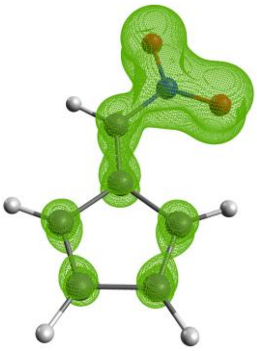

(p) NO_2-H

Table S4. Differential EDDB maps, illustrating the changes in global electron delocalization due to exocyclic Y substitution (in monosubstituted systems) or endocyclic X (in disubstituted systems), isovalue = + (green) or – (red) 0.005 electrons. Below figures details on which EDDB function was subtracted from which for each system.

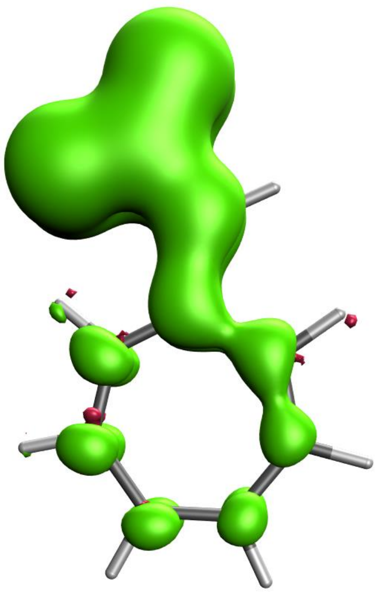

NO_2-H;
$EDDB_G$ (NO_2-H) – $EDDB_G$ (pentafulvene)

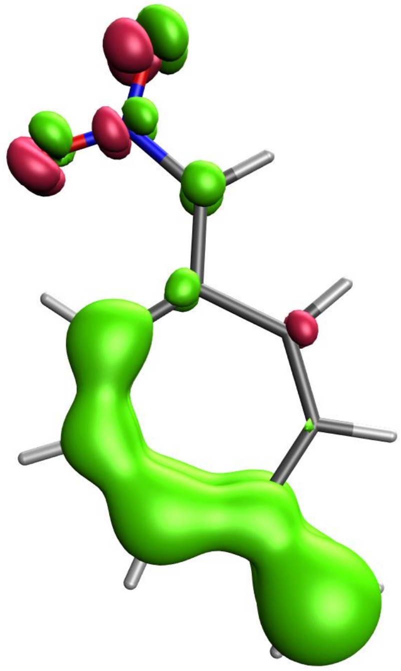

trans NO_2-NH_2-γ
$EDDB_G$ (trans NO_2-NH_2-γ) – $EDDB_G$ (NO_2-H)

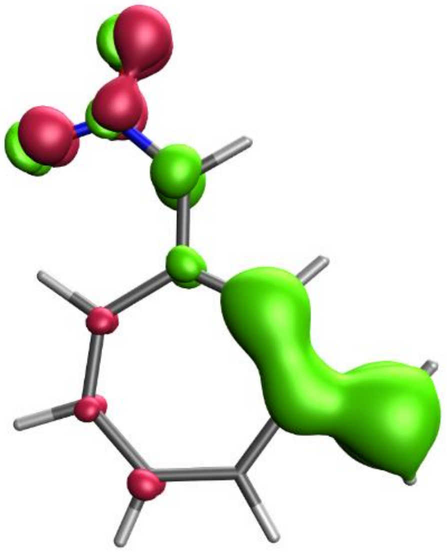

trans NO_2-NH_2-β
$EDDB_G$ (trans NO_2-NH_2-β) – $EDDB_G$ (NO_2-H)

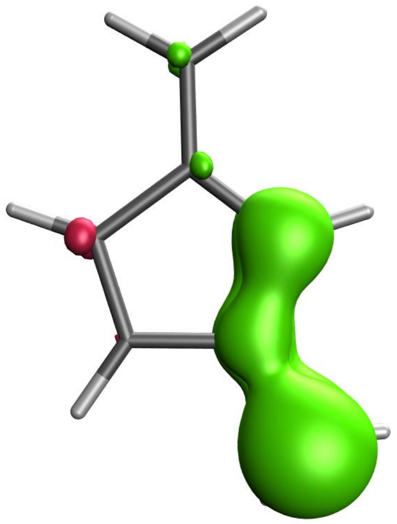

(p) H-NH_2
$EDDB_G$ [(p) H-NH_2] – $EDDB_G$ (pentafulvene)

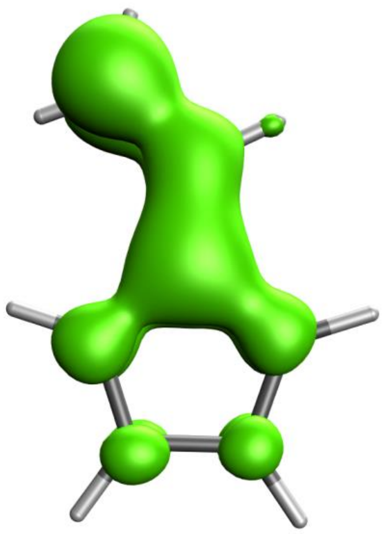

(p) NH_2-H
$EDDB_G$ [(p) NH_2-H] – $EDDB_G$ (pentafulvene)

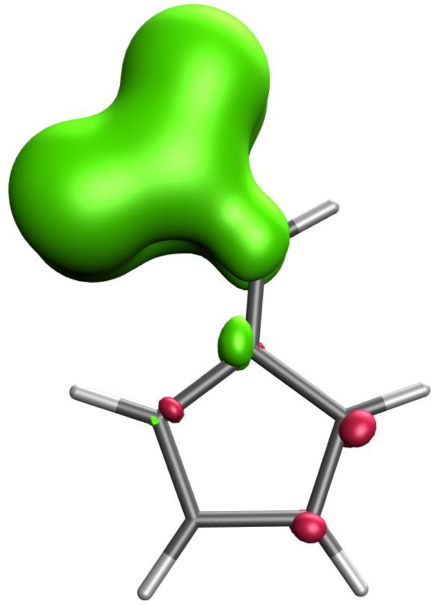

(p) NO_2-H
$EDDB_G$ [(p) NO_2-H] – $EDDB_G$ (pentafulvene)

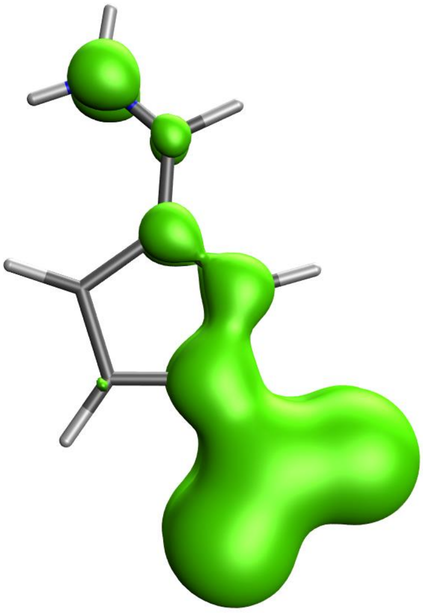

(p) trans NH_2-NO_2-β
$EDDB_G$ [(p) trans NH_2-NO_2-β] – $EDDB_G$ [(p) NH_2-H]

Table S5. Isosurfaces of the $EDDB_P(\pi)$ function (isovalue=0.005), representing the π-electron cyclic delocalization in hepta- and pentafulvene derivatives.

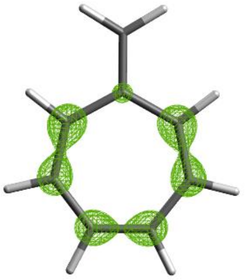

heptafulvene

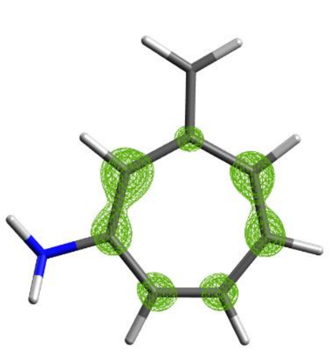

H-NH_2-β

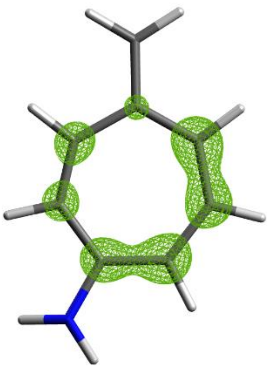

H-NH_2-γ

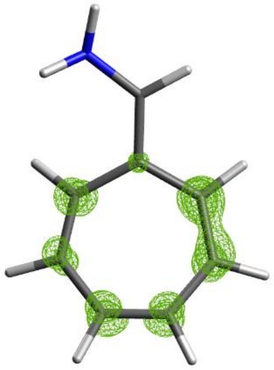

NH_2-H

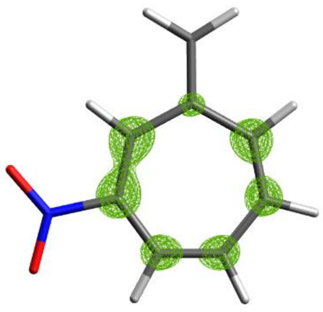

H-NO_2-β

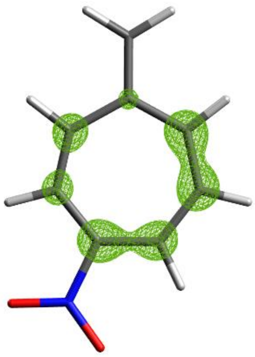

H-NO_2-γ

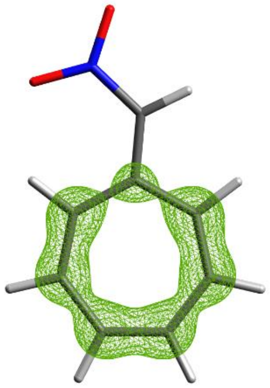

NO_2-H

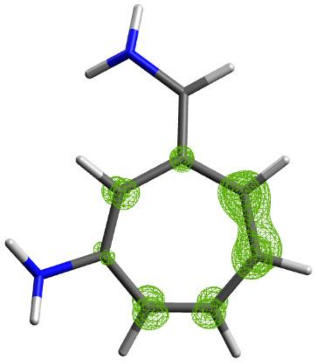

cis NH_2-NH_2-β

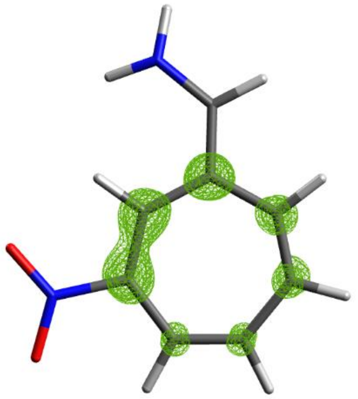

cis NO_2-NH_2-β

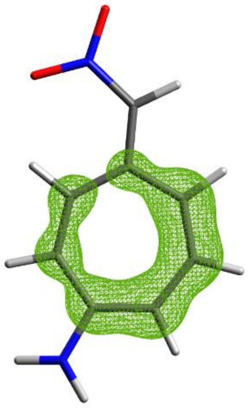

cis NO_2-NH_2-γ

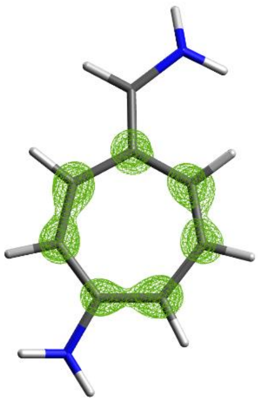

trans NH_2-NH_2-β

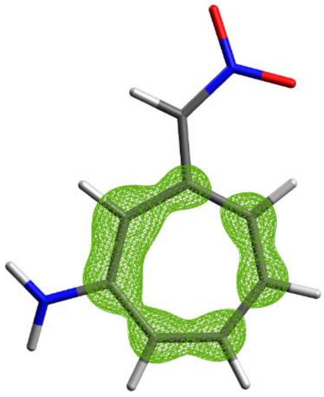

trans NO_2-NH_2-β

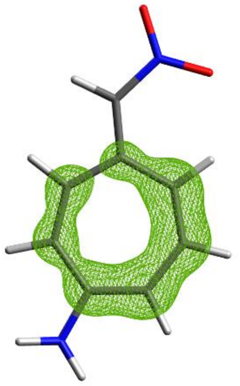

trans NO_2-NH_2-γ

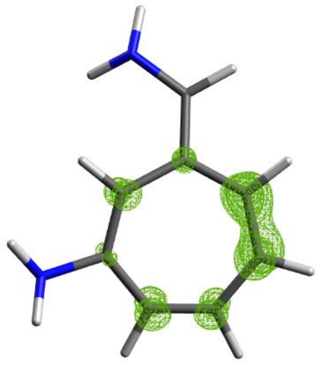

cis NH_2-NO_2-β

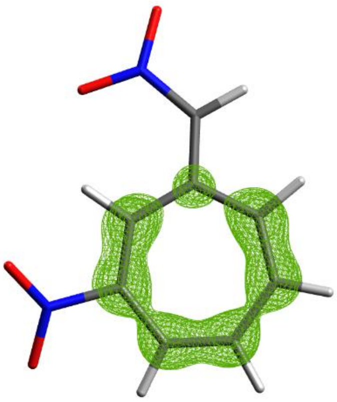

cis NO_2-NO_2-β

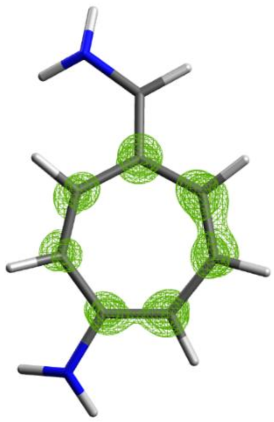

cis NH_2-NH_2-γ

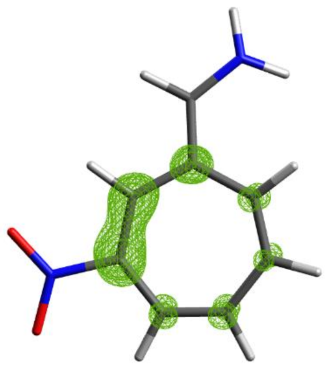

trans NH_2-NO_2-β

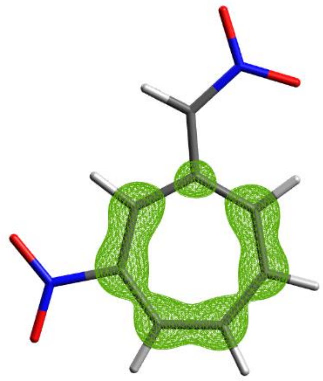

trans NO_2-NO_2-β

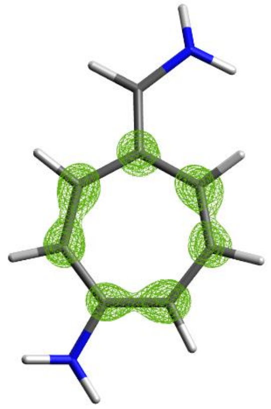

trans NH_2-NH_2-γ

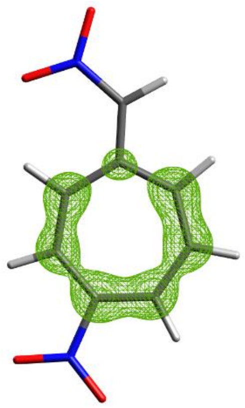

cis NO_2-NO_2-γ

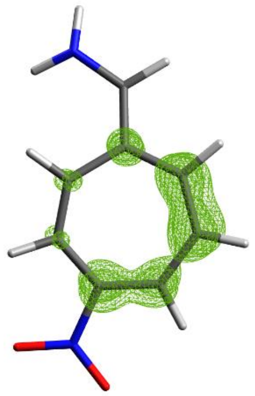

cis NH_2-NO_2-γ

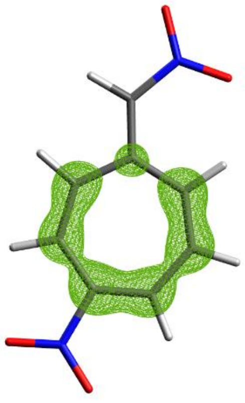

trans NO_2-NO_2-γ

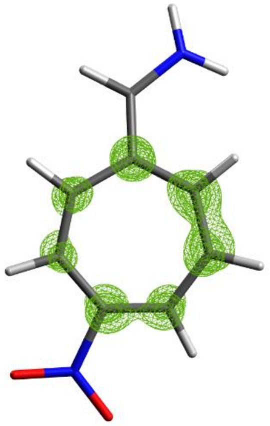

trans NH_2-NO_2-γ

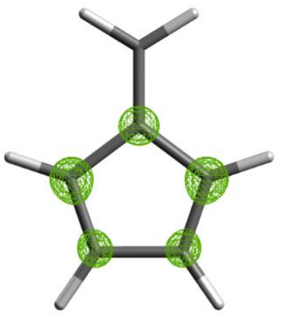

pentafulvene

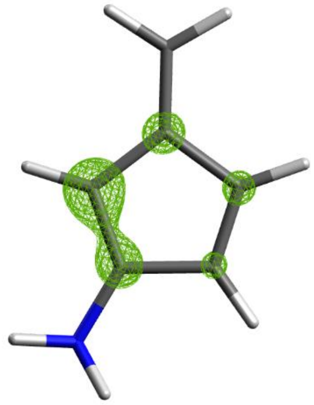

(p) H-NH_2-β

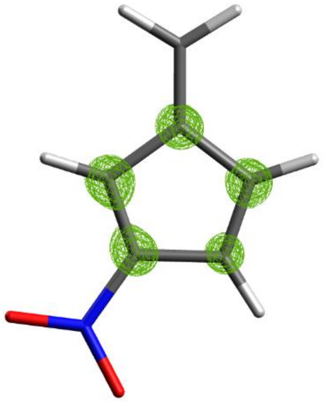

(p) H-NO_2-β

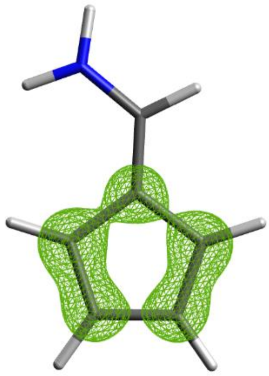

(p) NH_2-H

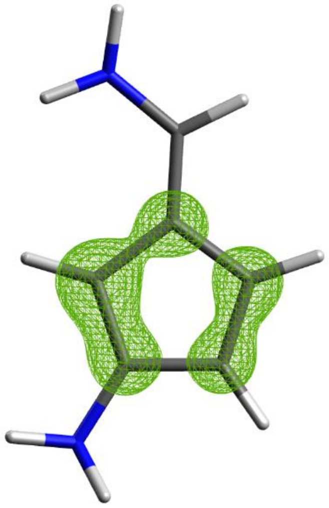

(p) cis NH_2-NH_2-β

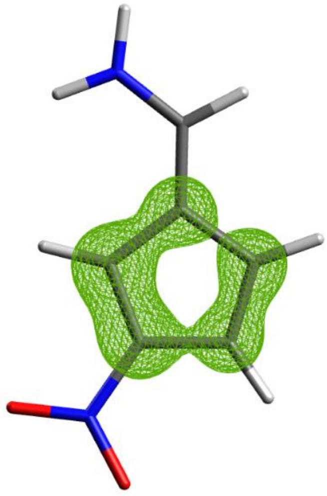

(p) cis NH_2-NO_2-β

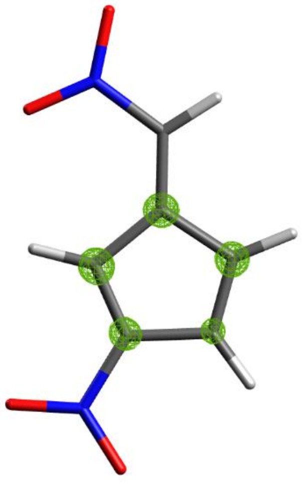

(p) cis NO_2-NO_2-β

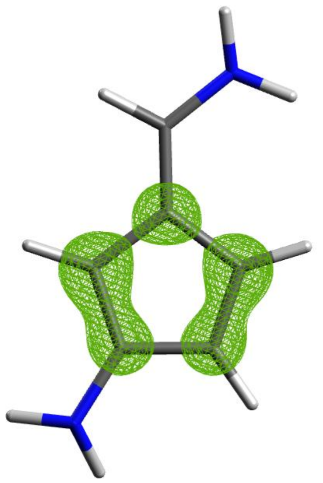

(p) trans NH_2-NH_2-β

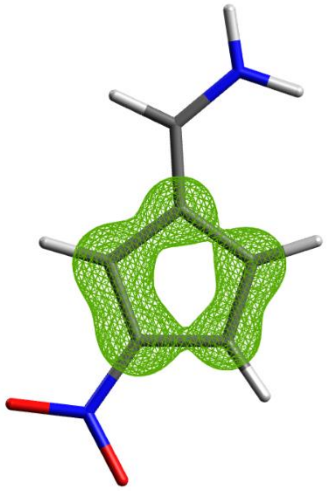

(p) trans NH_2-NO_2-β

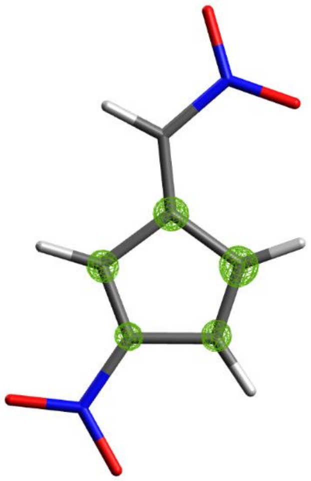

(p) trans NO_2-NO_2-β

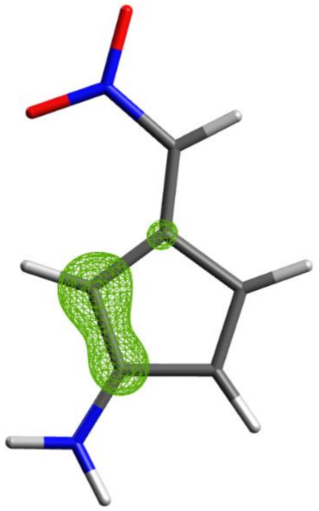

(p) cis NO_2-NH_2-β

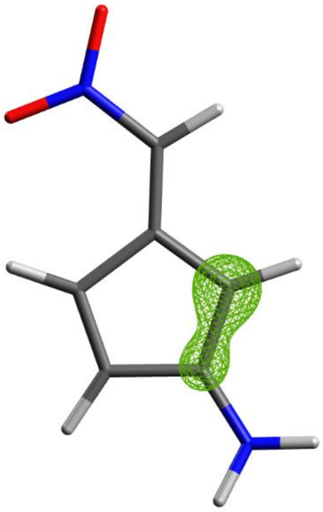

(p) trans NO_2-NH_2-β

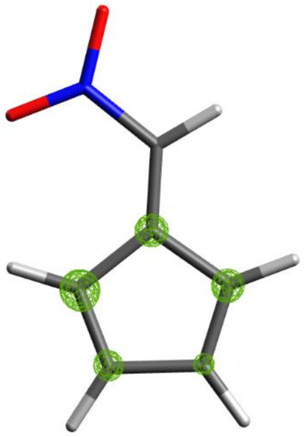

(p) NO_2-H

Table S6. Bond lengths (in Å) in studied penta- and heptafulvene derivatives. B3LYP/6-311++G(d,p) results.

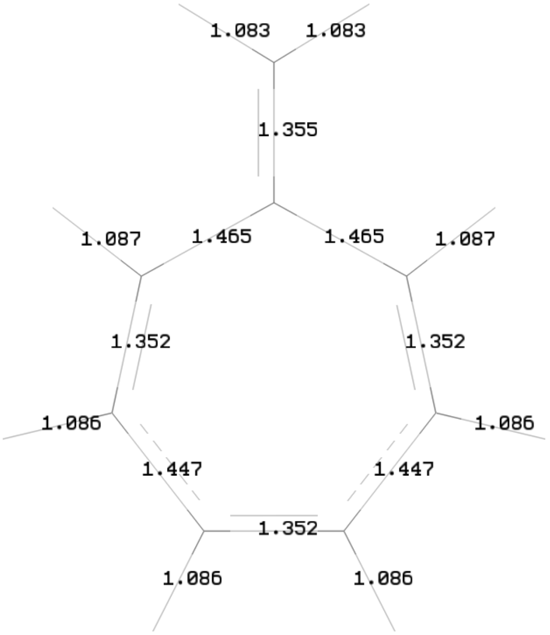

heptafulvene

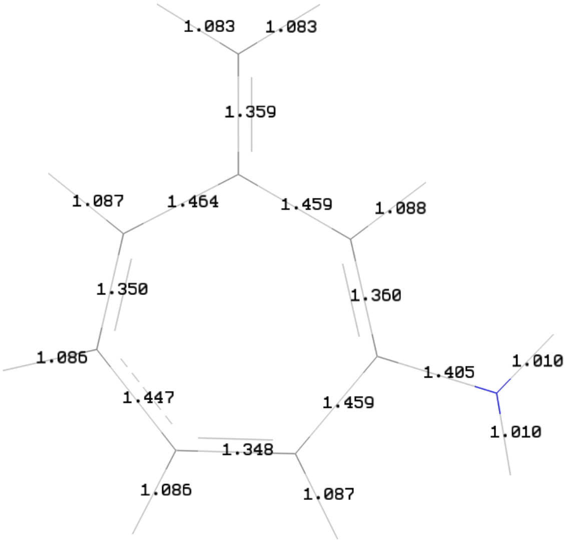

H-NH$_2$-β

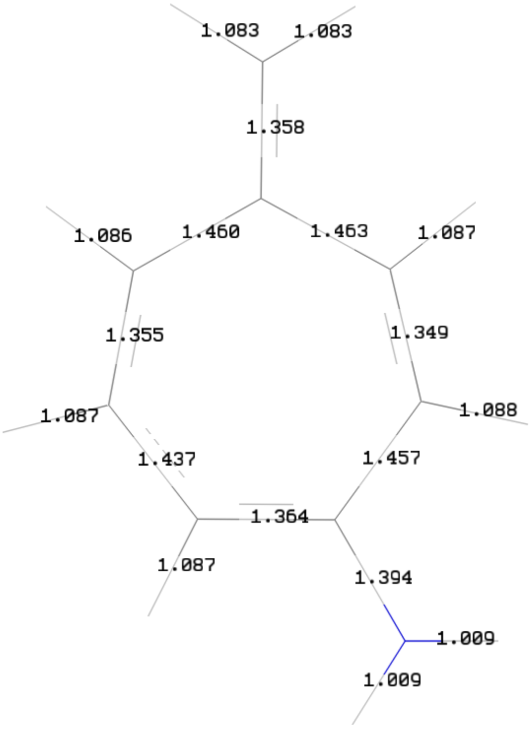

H-NH$_2$-γ

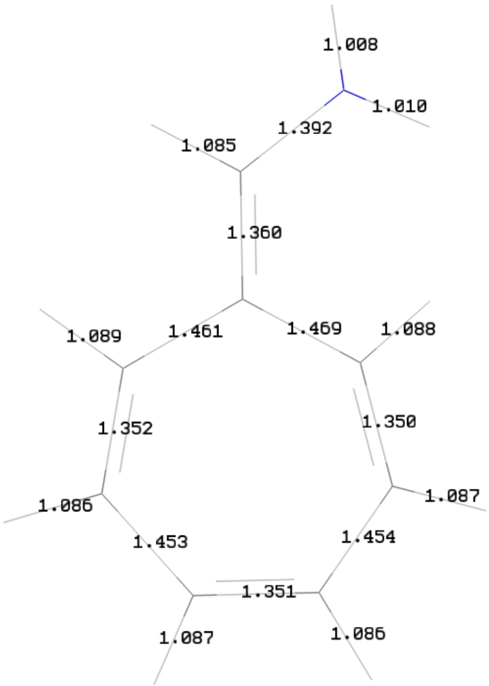

NH$_2$-H

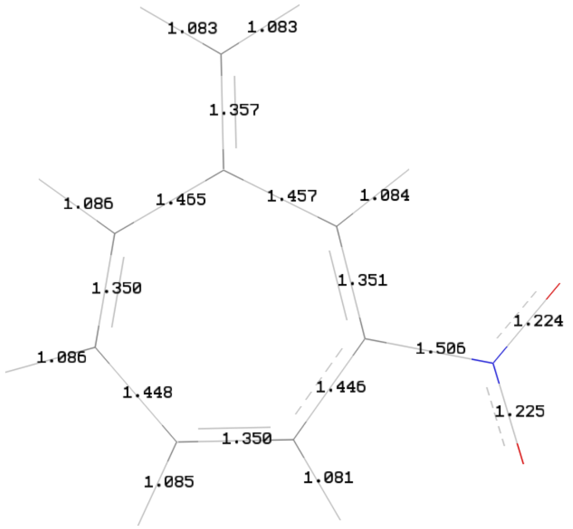

H-NO$_2$-β

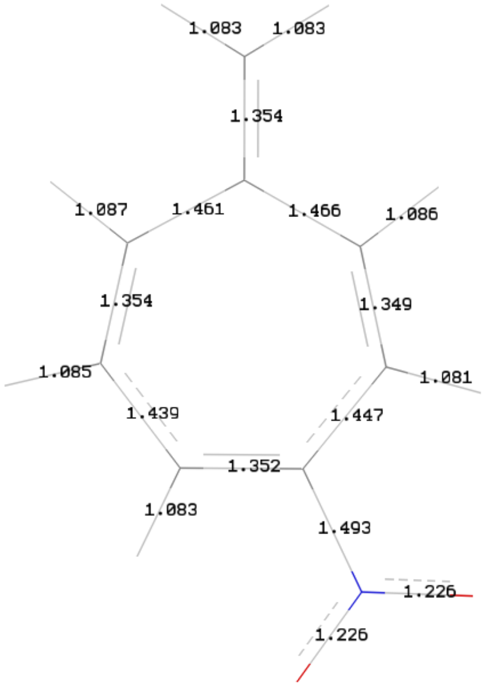

H-NO$_2$-γ

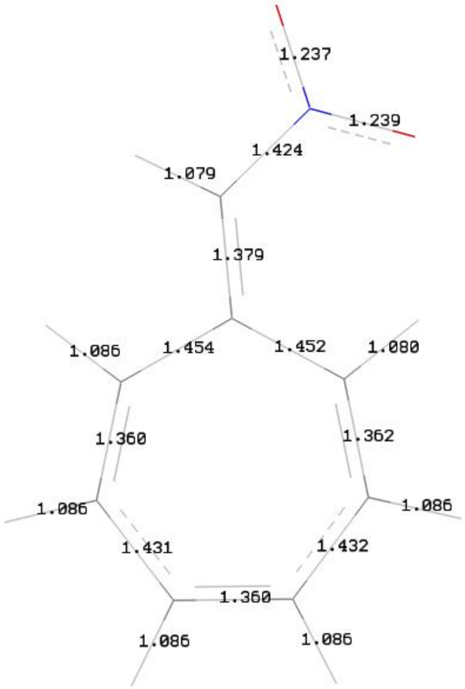

NO$_2$-H

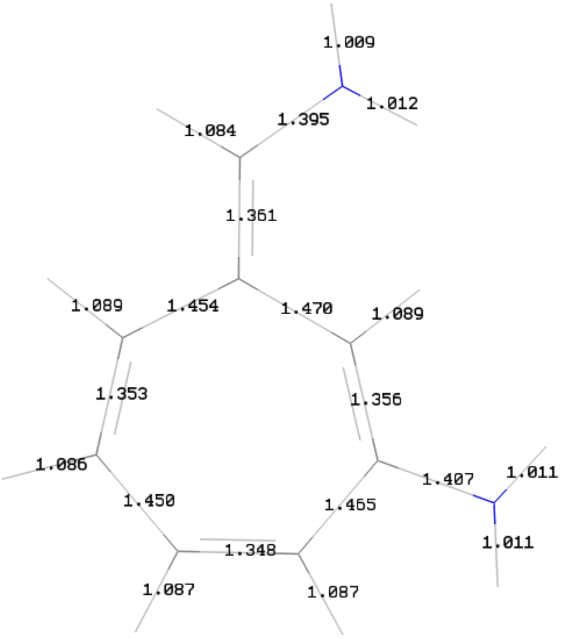

cis NH$_2$-NH$_2$-β

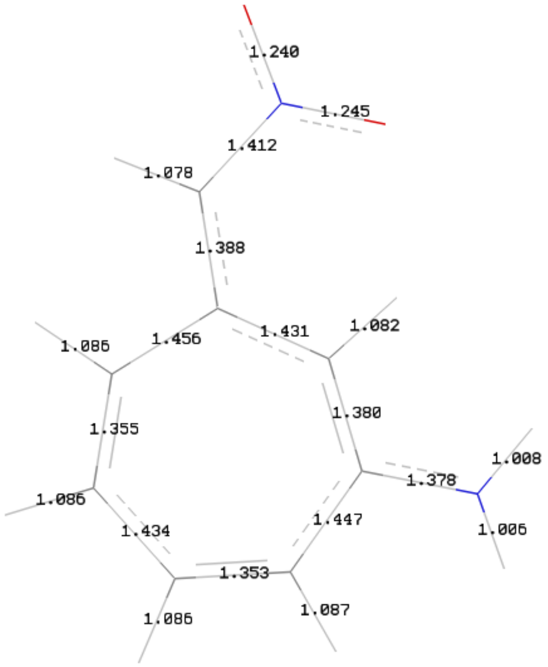

cis NO_2-NH_2-β

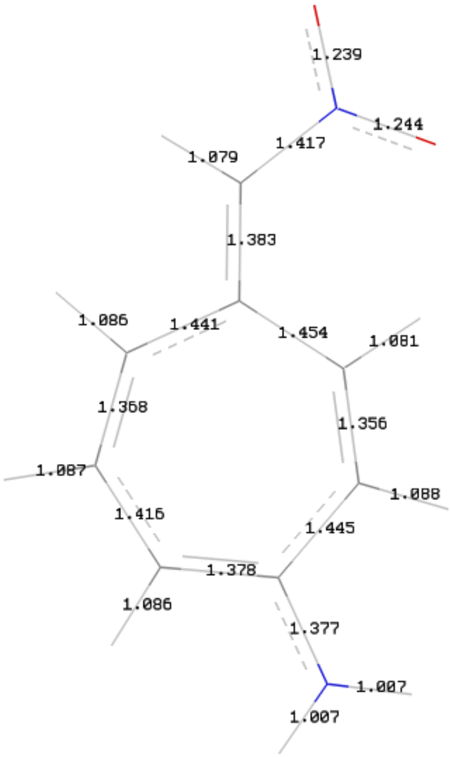

cis NO_2-NH_2-γ

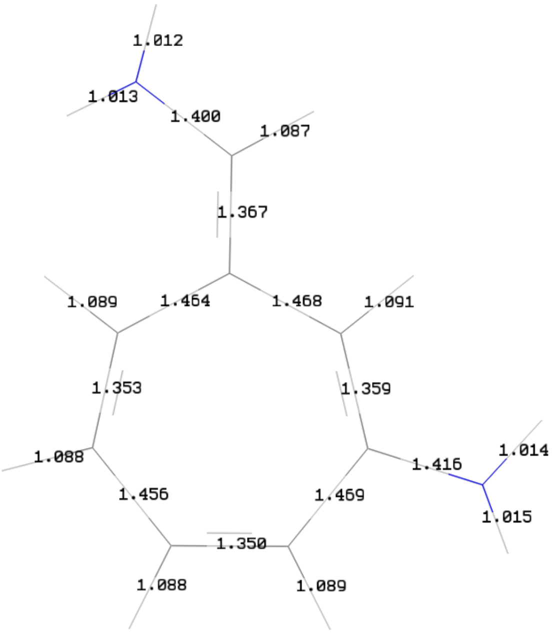

trans NH_2-NH_2-β

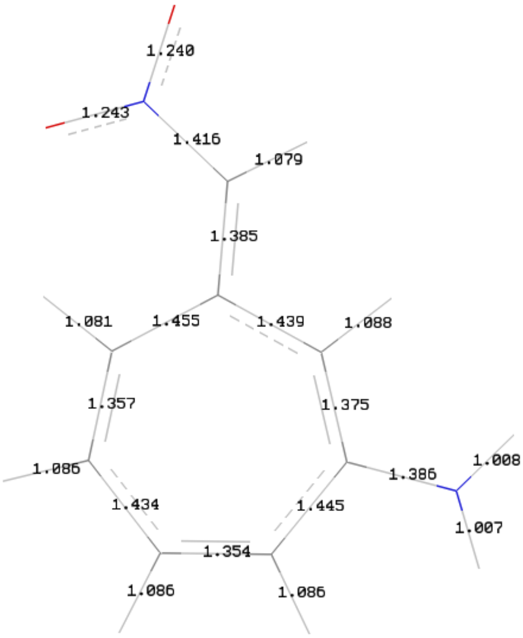

trans NO_2-NH_2-β

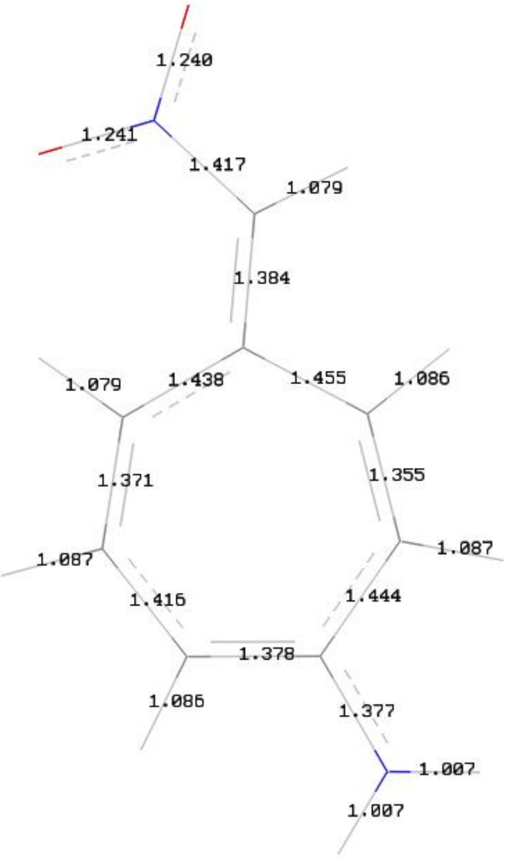

trans NO_2-NH_2-γ

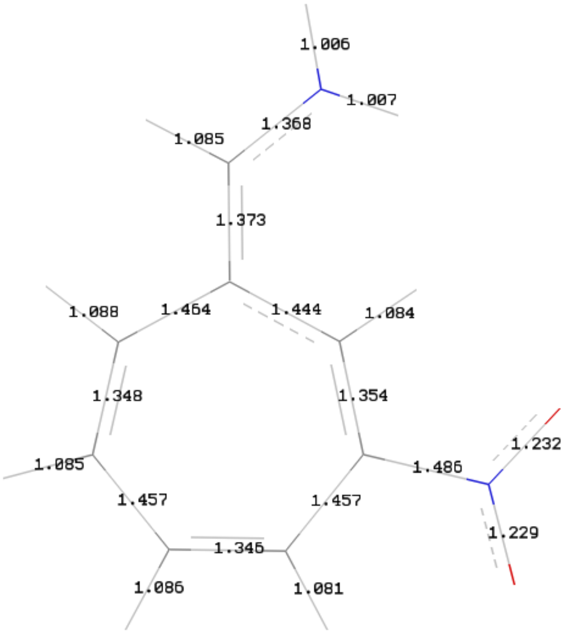

cis NH_2-NO_2-β

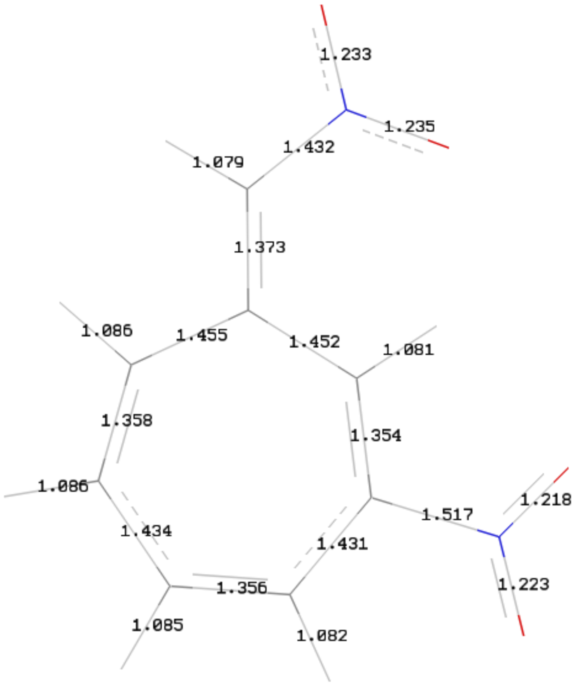

cis NO_2-NO_2-β

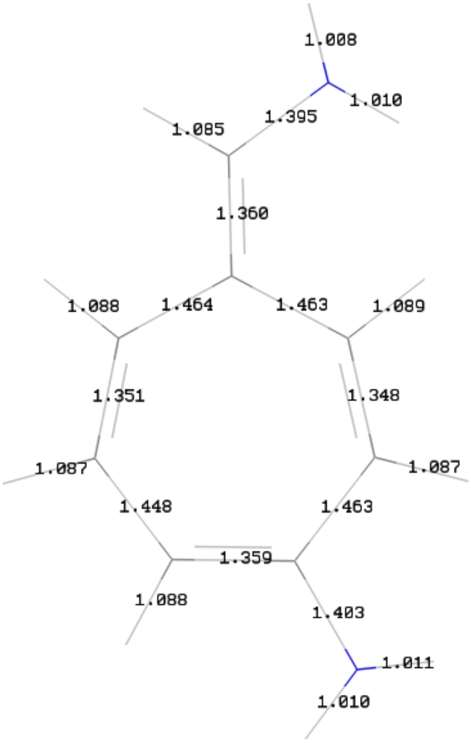

cis NH_2-NH_2-γ

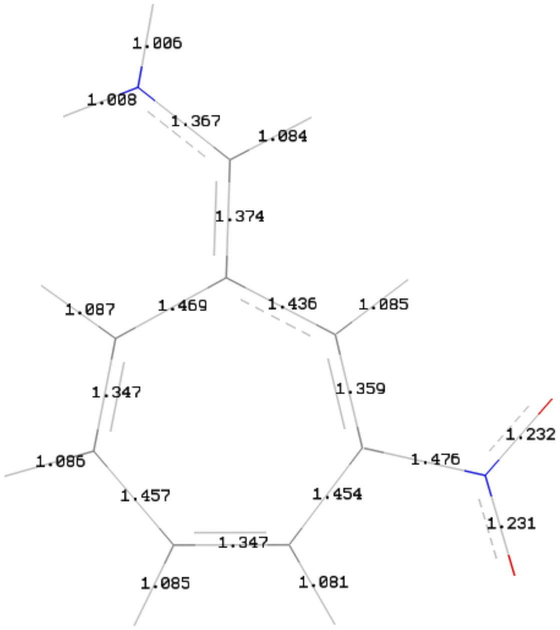

trans NH_2-NO_2-β

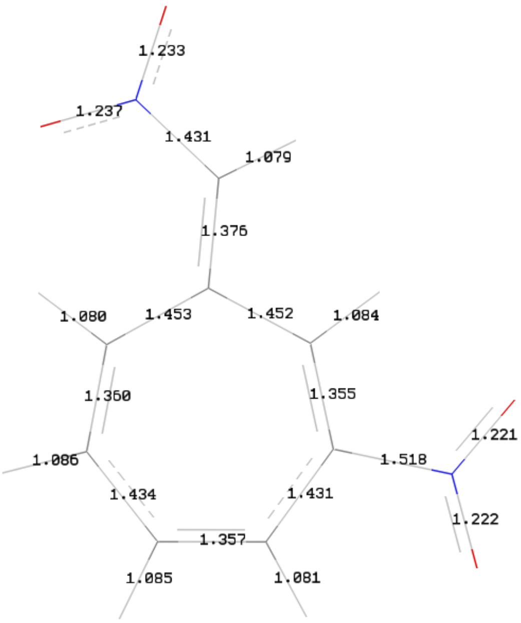

trans NO_2-NO_2-β

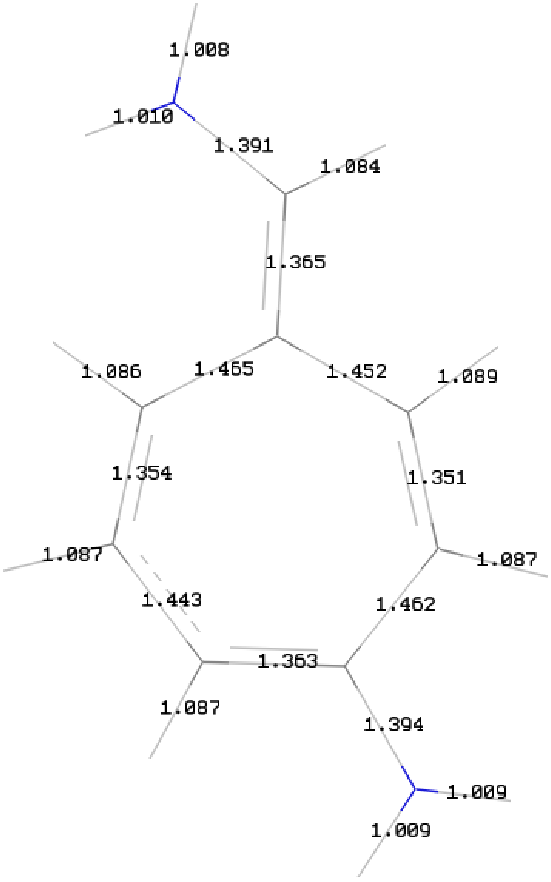

trans NH_2-NH_2-γ

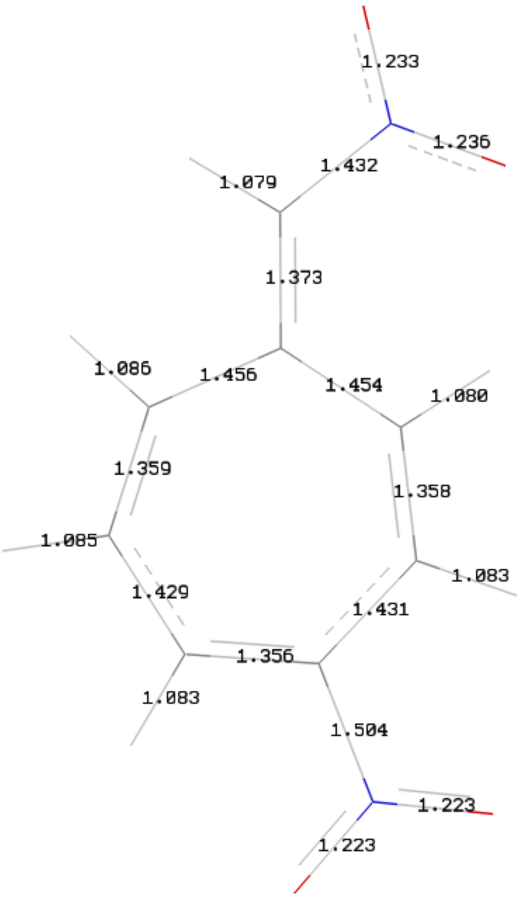

cis NO_2-NO_2-γ

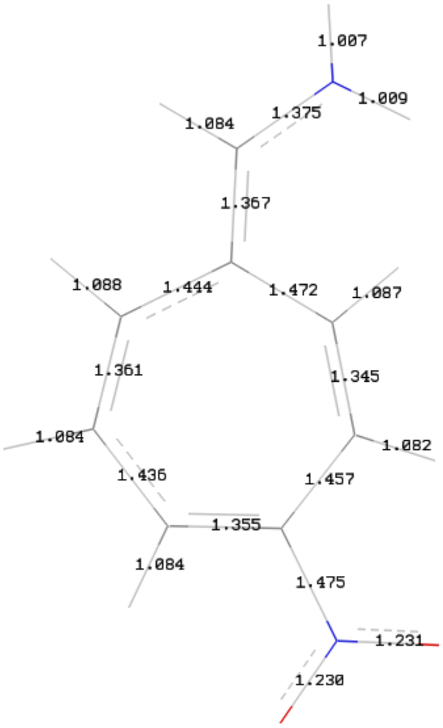

cis NH_2-NO_2-γ

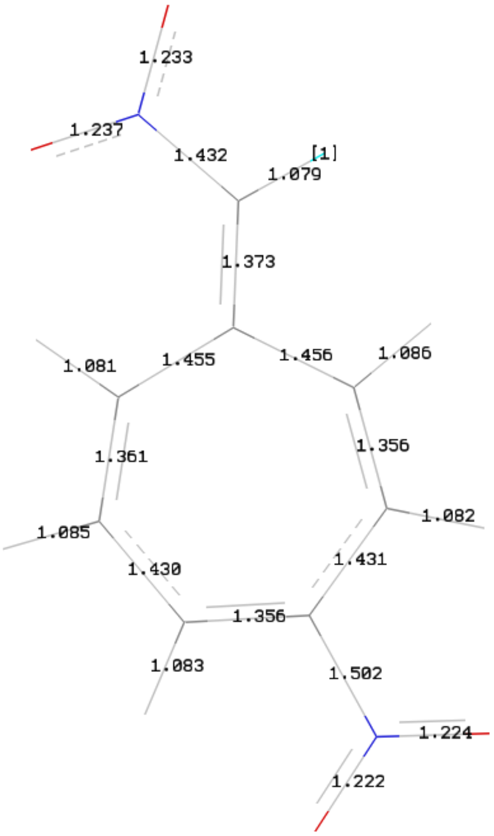

trans NO_2-NO_2-γ

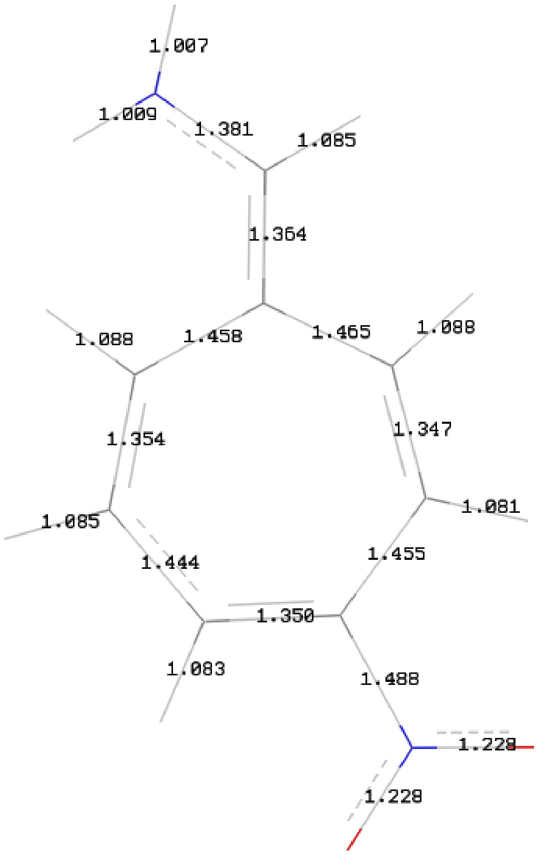

trans NH_2-NO_2-γ

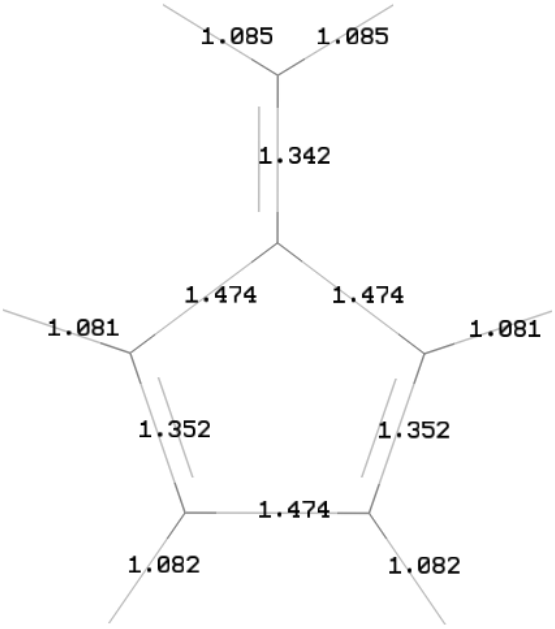

pentafulvene

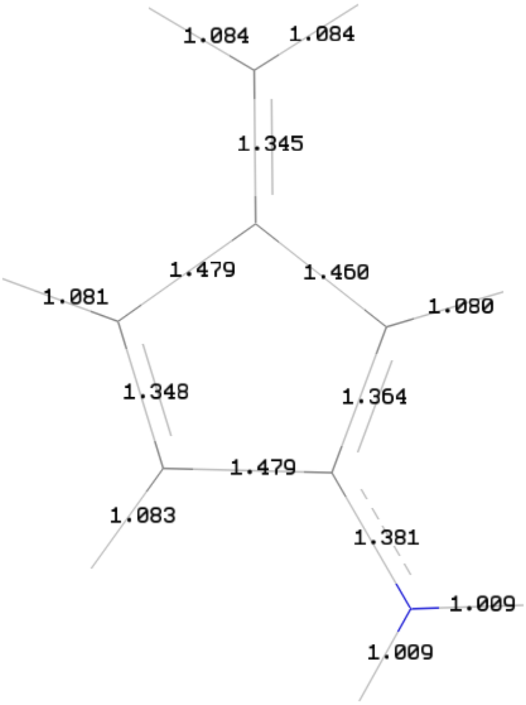

(p) H-NH_2-β

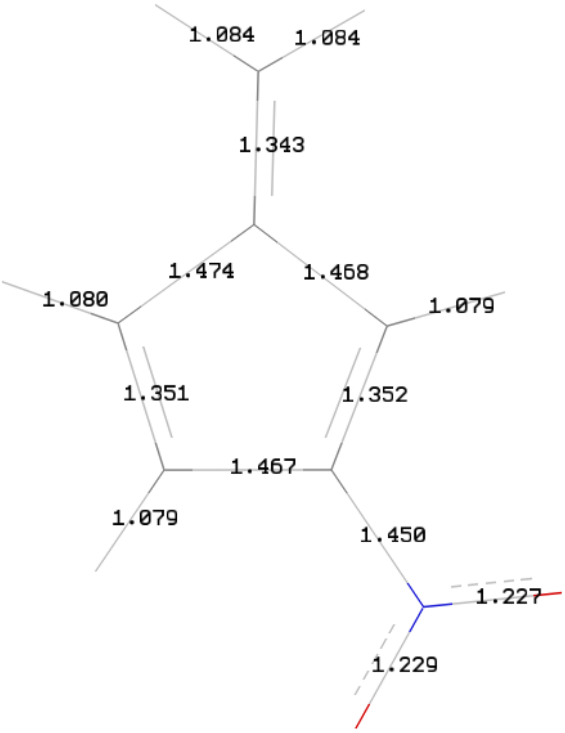

(p) H-NO_2-β

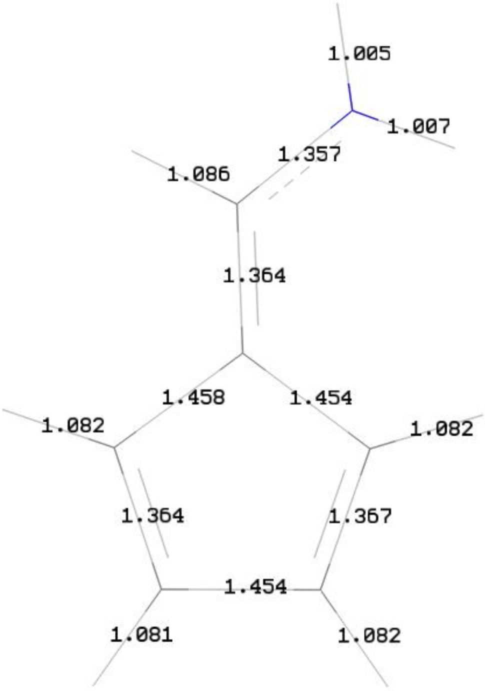

(p) NH_2-H

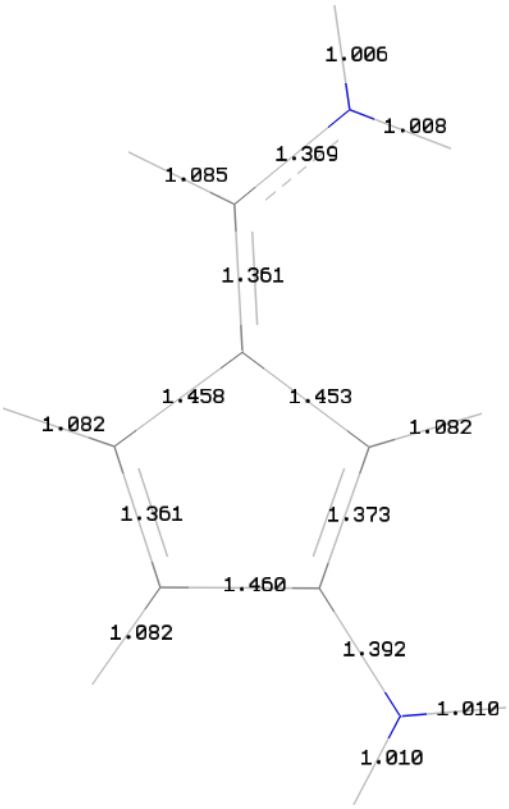

(p) cis NH_2-NH_2-β

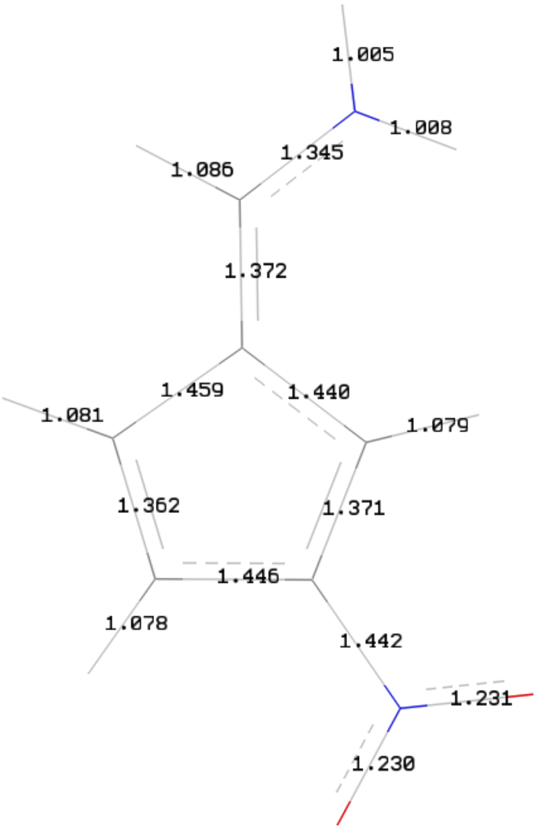

(p) cis NH_2-NO_2-β

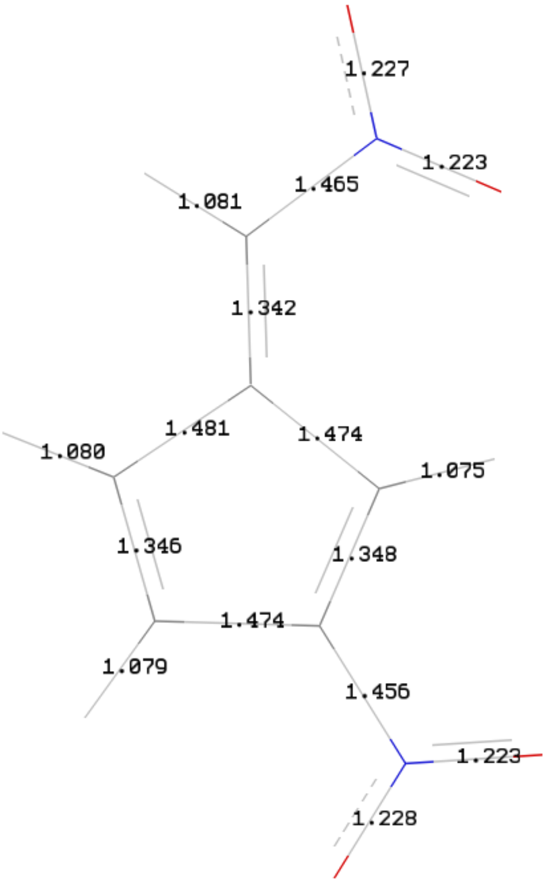

(p) cis NO_2-NO_2-β

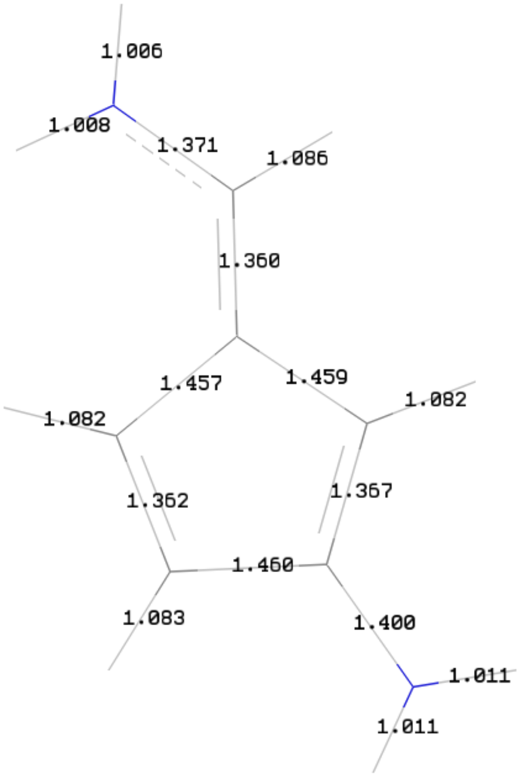

(p) trans NH_2-NH_2-β

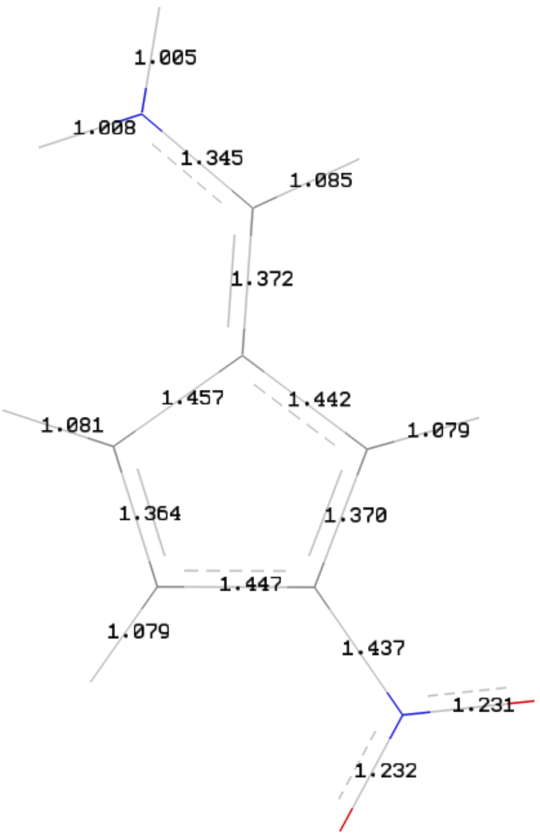

(p) trans NH_2-NO_2-β

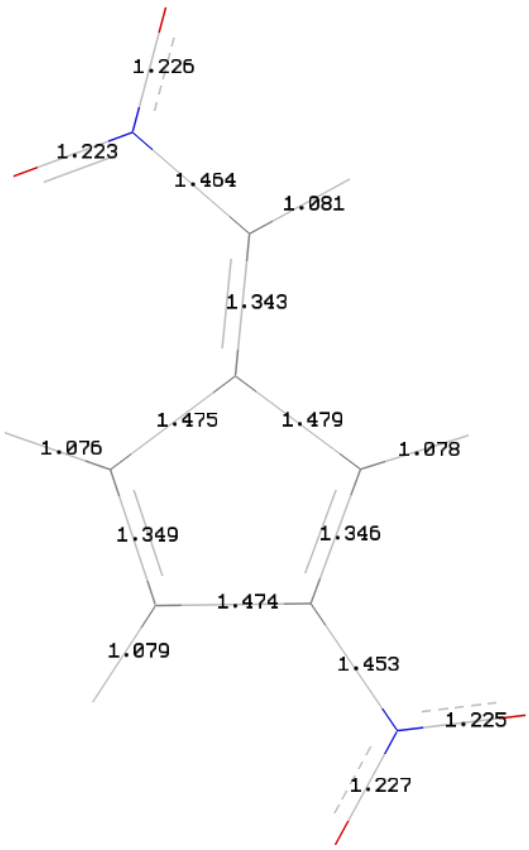

(p) trans NO_2-NO_2-β

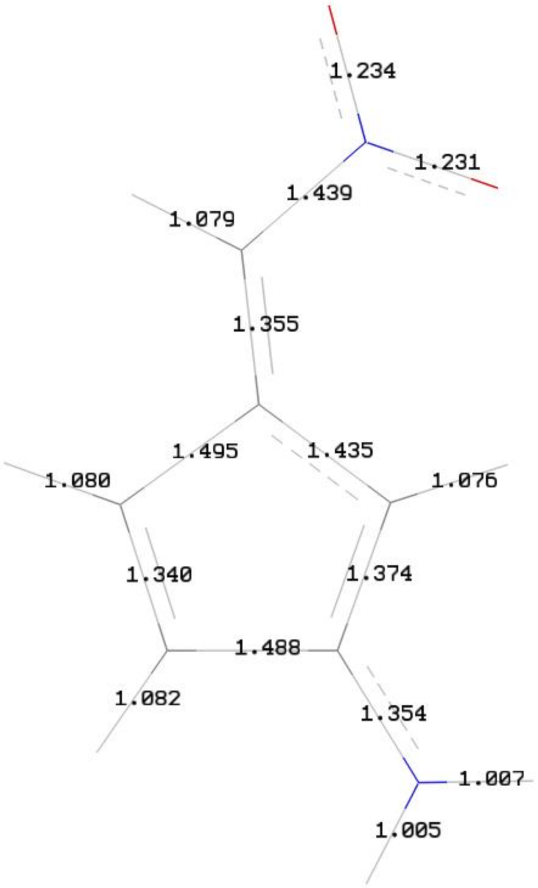

(p) cis NO_2-NH_2-β

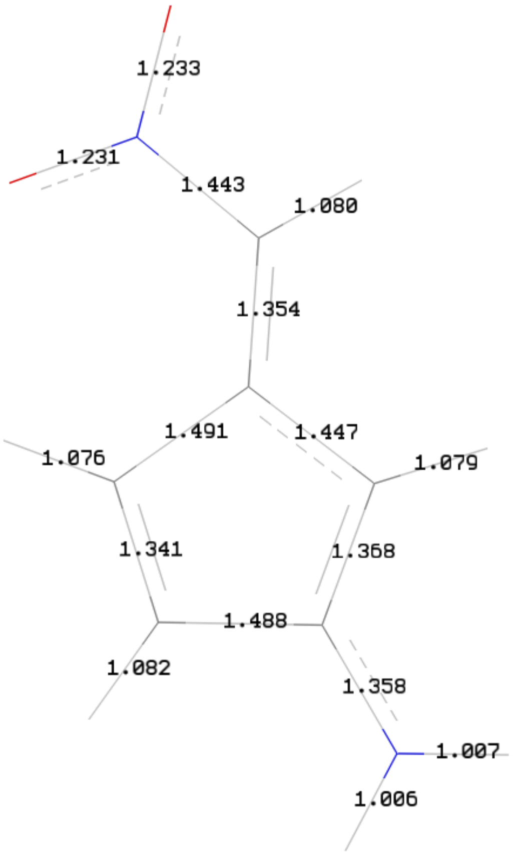

(p) trans NO_2-NH_2-β

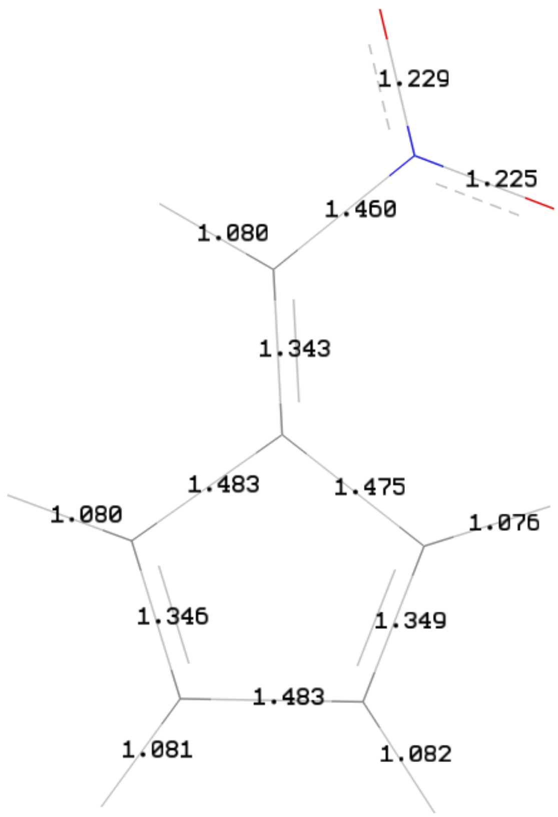
1.229
1.225
1.460
1.080
1.343
1.483
1.475
1.080
1.076
1.346
1.349
1.483
1.081
1.082

(p) NO_2-H

References

(1) Frisch, M. J.; Trucks, G. W.; Schlegel, H. B.; Scuseria, G. E.; Robb, M. A.; Cheeseman, J. R.; Scalmani, G.; Barone, V.; Petersson, G. A.; Nakatsuji, H.; Li, X.; Caricato, M.; Marenich, A. V.; Bloino, J.; Janesko, B. G.; Gomperts, R.; Mennucci, B.; Hratchian, H. P.; Ortiz, J. V.; Izmaylov, A. F.; Sonnenberg, J. L.; Williams-Young, D.; Ding, F.; Lipparini, F.; Egidi, F.; Goings, J.; Peng, B.; Petrone, A.; Hender-son, T.; Ranasinghe, D.; Zakrzewski, V. G.; Gao, J.; Rega, N.; Zheng, G.; Liang, W.; Hada, M.; Ehara, M.; Toyota, K.; Fukuda, R.; Hasegawa, J.; Ishida, M.; Nakajima, T.; Honda, Y.; Kitao, O.; Nakai, H.; Vreven, T.; Throssell, K.; Montgomery, J. A., Jr.; Peralta, J. E.; Ogliaro, F.; Bearpark, M. J.; Heyd, J. J.; Brothers, E. N.; Kudin, K. N.; Staroverov, V. N.; Keith, T. A.; Kobayashi, R.; Normand, J.; Raghavachari, K.; Rendell, A. P.; Burant, J. C.; Iyengar, S. S.; To-masi, J.; Cossi, M.; Millam, J. M.; Klene, M.; Adamo, C.; Cammi, R.; Ochterski, J. W.; Martin, R. L.; Morokuma, K.; Farkas, O.; For-esman, J. B.; Fox, D. J. Gaussian 16, Revision A.03, 2016.

(2) Krishnan, R.; Binkley, J. S.; Seeger, R.; Pople, J. A. Self-consistent Molecular Orbital Methods. XX. A Basis Set for Correlated Wave Functions. *J. Chem. Phys.* **1980**, *72* (1), 650–654.

(3) Kruszewski, J.; Krygowski, T. M. Definition of Aromaticity Basing on the Harmonic Oscillator Model. *Tetrahedron Lett.* **1972**, *13* (36), 3839–3842.

(4) Krygowski, T. M. Crystallographic Studies of Inter- and Intramolecular Interactions Reflected in Aromatic Character of .Pi.-Electron Systems. *J. Chem. Inf. Comput. Sci.* **1993**, *33* (1), 70–78.

(5) Matito, E.; Salvador, P.; Duran, M.; Solà, M. Aromaticity Measures from Fuzzy-Atom Bond Orders (FBO). The Aromatic Fluctuation (FLU) and the Para-Delocalization (PDI) Indexes. *J. Phys. Chem. A* **2006**, *110* (15), 5108–5113.

(6) Mayer, I. Bond Order and Valence Indices: A Personal Account. *J. Comput. Chem.* **2007**, *28* (1), 204–221.

(7) Lu, T.; Chen, F. Multiwfn: A Multifunctional Wavefunction Analyzer. *J. Comput. Chem.* **2012**, *33* (5), 580–592.

(8) Szczepanik, D. W.; Andrzejak, M.; Dyduch, K.; Żak, E.; Makowski, M.; Mazur, G.; Mrozek, J. A Uniform Approach to the Description of Multicenter Bonding. *Phys. Chem. Chem. Phys.* **2014**, *16* (38), 20514–20523.

(9) Szczepanik, D. W.; Andrzejak, M.; Dominikowska, J.; Pawełek, B.; Krygowski, T. M.; Szatylowicz, H.; Solà, M. The Electron Density of Delocalized Bonds (EDDB) Applied for Quantifying Aromaticity. *Phys. Chem. Chem. Phys.* **2017**, *19* (42), 28970–28981.

(10) Cyrański, M. K. Energetic Aspects of Cyclic Pi-Electron Delocalization: Evaluation of the Methods of Estimating Aromatic Stabilization Energies. *Chem. Rev.* **2005**, *105* (10), 3773–3811.

(11) Schleyer, P. V. R.; Pühlhofer, F. Recommendations for the Evaluation of Aromatic Stabilization Energies. *Org. Lett.* **2002**, *4* (17), 2873–2876.

(12) Chen, Z.; Wannere, C. S.; Corminboeuf, C.; Puchta, R.; Schleyer, P. V. R. Nucleus-Independent Chemical Shifts (NICS) as an Aromaticity Criterion. *Chem. Rev.* **2005**, *105* (10), 3842–3888.

(13) Stanger, A. NICS - Past and Present: NICS - Past and Present. *Eur. J. Org. Chem.* **2020**, *2020* (21), 3120–3127.

(14) Fallah-Bagher-Shaidaei, H.; Wannere, C. S.; Corminboeuf, C.; Puchta, R.; Schleyer, P. V. R. Which NICS Aromaticity Index for Planar π Rings Is Best? *Org. Lett.* **2006**, *8* (5), 863–866.

(15) Wolinski, K.; Hinton, J. F.; Pulay, P. Efficient Implementation of the Gauge-Independent Atomic Orbital Method for NMR Chemical Shift Calculations. *J. Am. Chem. Soc.* **1990**, *112* (23), 8251–8260.

(16) Sadlej-Sosnowska, N. Substituent Active Region – a Gate for Communication of Substituent Charge with the Rest of a Molecule: Monosubstituted Benzenes. *Chem. Phys. Lett.* **2007**, *447* (4–6), 192–196.

(17) Hirshfeld, F. L. Bonded-Atom Fragments for Describing Molecular Charge Densities. *Theoret. Chim. Acta* **1977**, *44* (2), 129–138.
